# Role of DNA-LL37 complexes in the activation of plasmacytoid dendritic cells and monocytes in subjects with type 1 diabetes

**DOI:** 10.1038/s41598-020-65851-y

**Published:** 2020-06-01

**Authors:** Darshan Badal, Devi Dayal, Gunjan Singh, Naresh Sachdeva

**Affiliations:** 1 Department of Pediatrics, Post Graduate Institute of Medical Education and Research (PGIMER), Chandigarh, India; 2 Department of Endocrinology, Post Graduate Institute of Medical Education and Research (PGIMER), Chandigarh, India

**Keywords:** Autoimmunity, Type 1 diabetes, Paediatric research

## Abstract

Initiation of type 1 diabetes (T1D) is marked by the infiltration of plasmacytoid dendritic cells (pDCs) and monocytes in pancreatic islets. Dying beta cells release self-DNA, which forms complexes with antimicrobial peptide, LL37, and its delayed clearance can activate pDCs and monocytes. Here, we studied the phenotypic effects of DNA-LL37 complexes on pDCs and monocytes in 55 recently diagnosed T1D and 25 healthy control (HC) subjects. Following *in vitro* stimulation with DNA-LL37 complexes, T1D group demonstrated higher frequency and mean fluorescence intensity (MFI) of pDCs expressing IFN-α. Similarly, the monocytes in T1D group showed an increase in MFI of IFN-α. Post-stimulation, an increase in the antigen presentation and co-stimulatory ability of pDCs and monocytes was observed in T1D group, as indicated by higher expression of HLA-DR, CD80 and CD86. Upon co-culture, the stimulated monocytes and pDCs, particularly in the T1D group were able to further activate autologous CD4 + T cells, with increase in expression of CD69 and CD71. Finally, in a transwell assay, the stimulated pDCs and monocytes induced an increase in apoptosis of 1.1B4 beta cells. Additionally, we observed reduced expression of indoleamine 2,3-dioxygenase 1 (IDO1) in pDCs and monocytes of T1D subjects. Our results suggest that DNA-LL37 complexes activate pDCs and monocytes towards a proinflammatory phenotype during pathogenesis of T1D.

## Introduction

Type 1 diabetes (T1D) is an autoimmune disease manifested by chronic hyperglycaemia due to the destruction of pancreatic beta cells by host immune cells. The disease is characterized by the infiltration of mononuclear cells, including CD4 + T cells, CD8 + T cells, B cells, plasmacytoid dendritic cells (pDCs) and macrophages, of which CD8 + T cells are predominant^1–3^.

pDCs contribute to the pathogenesis of autoimmune diabetes through the secretion of type 1 interferons (IFN), like IFN-α which plays a role in the transition of prediabetes stage to full-blown diabetes by facilitating the infiltration of autoreactive T cells^4–8^. It has been reported that pDCs accumulate in the islets of non-obese diabetic (NOD) mice as early as 2 weeks of age and get activated via toll-like receptor (TLR)9 by DNA-cathelicidin-related antimicrobial peptide (CRAMP) complexes, leading to the production of IFN-α and induction of autoimmune diabetes^9^. Newly diagnosed subjects with T1D and those at high risk, have a higher frequency of pDCs with enhanced IFN-α production following *in vitro* stimulation of peripheral blood mononuclear cells (PBMCs) with influenza viruses. Small DNA molecules like CpG 2216 mediate IFN-α production by pDCs, which was found to be highest in first degree relatives of subjects with T1D^10^. In addition to pDCs, the role of activated macrophages in beta cell destruction has been demonstrated in both *in vivo* and *in vitro* studies^11,12^. Martin *et al*. (2008) provided evidence that monocytes are capable of destroying beta cells, even in the absence of mature T and B cells^13^. Ren *et al*. (2017) observed that intermediate CD14 + CD16 + monocytes of subjects with T1D had better antigen presentation capability as indicated by the increase in the expression of human leukocyte antigen (HLA)-DR and CD86^14^. However, the initial events that cause the activation of pDCs and monocytes in T1D are not yet clear.

Beta cells are fragile in nature either due to genetic predisposition, viral infections, or high rates of insulin turnover, making them susceptible to endoplasmic reticulum (ER) stress resulting in beta cell apoptosis^15–19^. The initial infiltration and proximity of pDCs and monocytes make them an ideal candidate to pick up and process alarmin molecules like self-DNA released from dying beta cells^20,21^. Self-DNA is normally non-immunogenic due to its rapid degradation and seclusion of DNA recognizing receptors inside the cells^22^. However, binding of self-DNA to neutrophil-derived antimicrobial peptide, LL37, can break tolerance to self-DNA by protecting and transporting it into endosomal compartments of pDCs and monocytes as seen in other diseases like systemic lupus erythematosus (SLE) and atherosclerosis^23–27^. Cathelicidin LL37 antimicrobial peptides are expressed by several leukocytes, epithelial cells and are known for their antibacterial and immune cell attracting roles^28^. Contrarily, a study by Merkle *et al*. (2015) demonstrated that DNA internalized by LL37 in non-immune cells like human microvascular endothelial cells inhibits pro-inflammatory responses^29^. Whether LL37 aided transport of self-DNA into monocytes and pDCs can lead to activation of these cells and increased type I IFN in subjects with T1D, is still unknown. In addition to the inflammatory responses, pDCs and monocytes can also skew immune responses towards tolerance by mechanisms, like indoleamine 2,3-dioxygenase (IDO) production, an important enzyme in tryptophan catabolism, impairment of which leads to defective tolerance and even its transient upregulation downregulates T1D^30–32^. In response to inflammation, IDO is known to curtail T cell proliferation by promoting T cell anergy and induction of regulatory T cells (Tregs)^33^. Several studies have investigated the role of IDO in T1D and found that loss of IDO precedes islet destruction and induction of IDO in islets confers beta cell protection in a TLR9 driven manner^34–37^. Another study suggested a tolerogenic role of pDCs through early infiltration in islets and IDO production to curtail effector T cell responses^38^.

There is a lack of literature on the interaction of pDCs and monocytes with dying beta cells and the resulting changes in their phenotype, especially when viral infection alone cannot explain the infiltration of pDCs and monocytes with an increase in IFN-α signature. In view of this, the present work was aimed to study three aspects of the interaction of DNA immune complexes with pDCs and monocytes. At first, we investigated whether the uptake of DNA-LL37 complexes confers a proinflammatory phenotype to pDCs and monocytes by either increasing IFN-α production or enhancing their antigen presentation capacity in terms of increased expression of HLA-DR, CD80 and CD86. Next, we studied the activation of CD4 + T cells by pDCs and monocytes stimulated with preproinsulin and DNA-LL37 complexes, by analysing CD69 and CD71 expression. Finally, we analysed the ability of activated pDCs and monocytes to induce beta cell killing *in vitro*. Our study revealed a mechanism whereby self-DNA upon binding with LL37 triggers an IFN-α response leading to activation of pDCs and monocytes, which further activate CD4 + T cells and cause beta cell apoptosis.

## Material and Methods

### Subjects

A total of 55 subjects with recently diagnosed T1D (< 6 months) and 25 healthy control (HC) subjects were recruited from the departments of Pediatrics and Endocrinology at the Post Graduate Institute of Medical Education and Research (PGIMER), Chandigarh, India. Diabetes was diagnosed as per American Diabetes Association (ADA) criteria^39^. Inclusion criteria for T1D were the presence of autoantibodies to glutamic acid decarboxylase (GAD)65 or islet antigen-2 (IA-2) or insulin, whereas, exclusion criteria included, presence of infectious diseases, anaemia (Hb < 8.0 g⁄dl), any acute illness, other autoimmune diseases (including celiac disease), lymphomas, psychiatric illness, and pregnancy. The subjects were recruited after going through their clinical history. The study was approved by the institutional ethics committee of PGIMER, Chandigarh. After obtaining informed consent, fasting peripheral blood samples (15 ml) were obtained in heparinized vacutainers immediately after the diagnosis of T1D. Peripheral blood was used for the determination of fasting plasma C-peptide and haemoglobin A1c (HbA1c) (Table 1). The titres of anti-insulin (Orgentec Diagnostika, Mainz, Germany), anti-GAD65 and anti-IA-2 (RSR Limited, Cardiff, UK) autoantibodies in all subjects were analysed by ELISA as per manufactures’ instructions.

**Table 1:** Clinical characteristics of recruited subjects. Data is presented as mean (±SEM).

Characteristic	T1D	HC
Patients (*n*)	55	25
Age (years)	12.9 ± 0.55	19.78 ± 1.47
Sex (M/F)	31/24	16/9
Fasting plasma C-peptide (ng/mL)*	0.43 ± 0.05	2.61 ± 0.29
HbA1c (%)*	11.04 ± 0.37	5.21 ± 0.07
Anti-insulin antibodies (% subjects)	16/55 (29%)	0/25 (0%)
Anti-IA-2 antibodies (% subjects)	23/55 (42%)	0/25 (0%)
Anti-GAD65 antibodies (% subjects)	38/55 (69%)	1/25 (4%)
Triple antibody positive (% subjects)	10/55 (18%)	0/25 (0%)
Double antibody positive (GAD65 and IA-2, % subjects)	11/55 (20%)	0/25 (0%)
Double antibody positive (GAD65 and Insulin, % subjects)	6/55 (11%)	0/25 (0%)
Double antibody positive (IA-2 and Insulin, % subjects)	3/55 (5%)	0/25 (0%)

M/F, Male/female; HbA1c, Glycated haemoglobin; GAD, Glutamic acid decarboxylase; IA-2, Islet antigen 2.

* p < 0.01, T1D versus HC. Unpaired t-test was used to compare means between different groups.

The minimum detectable levels (with intra, inter-assay CV) for anti-insulin was 0.5 U/mL (3.1, 5.1%), anti-GAD65, 0.57 u/mL (6.4, 5.8%) and anti-IA-2, 0.95 u/mL (2.0, 4.3%).

### Cell isolation

PBMCs were isolated using Ficoll-Hypaque (1.077 g/L) (Sigma Aldrich, St. Louis, USA) by density gradient centrifugation and re-suspended in complete Roswell Park Memorial Institute (RPMI)-1640 [supplemented with 0.1% penicillin, streptomycin and 10% fetal bovine serum (FBS)] (Life technologies, Carlsbad, USA) for further downstream applications. pDCs were sorted by magnetic cell isolation using CD304 [blood dendritic cell antigen (BDCA)-4/Neuropilin-1] microbeads (Miltenyi Biotec GmBh, Bergisch Gladbach, Germany) according to the manufacturer’s instructions^40^. The purity of enriched pDCs was assessed by staining with human Fluorescein isothiocyanate (FITC) labelled BDCA-2 (clone AC144) (Miltenyi Biotec GmBh) and brilliant violet (BV)605 labelled CD123 (clone 7G3) (BD Biosciences, San Jose, USA) antibodies. Data was acquired using BD FACS Diva^TM^ software (BD Biosciences).

For monocytes, isolated PBMCs were seeded in serum-free complete RPMI media in 12 well tissue culture plates (BD Falcon, San Jose, USA) for 2–3 hours at 37°C in a humidified CO_2_ incubator. Adherent monocytes were isolated by the removal of non-adherent cells, followed by trypsinization for detachment of monocytes^41^. Human CD4 + CD25low/- T cells were obtained from PBMCs using human CD4 + CD25hi T cell isolation kit (after separation of CD4 + CD25 + T cells) (Stem Cell Technologies, Vancouver, Canada). CD8 + T cells were isolated from PBMCs using the EasySep™ Human CD8 + T cell isolation kit (Stem Cell Technologies, Vancouver, Canada).

### Generation of DNA-LL37 complexes and stimulation of pDCs and monocytes

Whole cell genomic DNA was isolated from PBMCs of each subject using the QiAmp DNA Mini Kit (Qiagen, Hilden, Germany). Self-DNA was incubated with a recombinant antimicrobial peptide, LL37 (AnaSpec, CA, USA), at different ratios in 1X phosphate buffer saline (PBS) (pH 7.4) at room temperature (RT) at different time points (5–60 minutes) for standardization of optimal formation of DNA-LL37 complexes. Finally, in all experiments, 1 µg of DNA was mixed in 5 µg of LL37 peptide in 20 µl PBS^42^, and multiple aliquots were formed for experiments and stored at −20 °C to avoid batch-to-batch variations. Free DNA was removed by DNase 1 (4 units in 2 µL, Sigma-Aldrich, USA) treatment for 30 minutes at RT and the reaction was stopped with 1 M NaCl. The DNA-LL37 complexes were then diluted in complete RPMI medium at a final concentration of 3 µg/100 µL and added to pDC and monocytes in cultures according to experimental requirement. In each experiment, genomic DNA used in the formation of DNA-LL37 complexes and cells (pDCs, monocytes, CD4 + T and CD8 + T cells) used for various assays, were obtained from the same subject.

#### Confirmation of DNA-LL37 complexes formation

The DNA-LL37 complex formation was confirmed by electrophoretic mobility shift assay (EMSA) using agarose gel and native polyacrylamide gel electrophoresis (PAGE) gel. The DNA-LL37 complexes were treated with DNase I as described before. For EMSA on agarose gel, the samples (DNase I-treated and untreated DNA-LL37 complexes and DNA alone) were mixed with DNA loading dye (Thermo Fisher Scientific, Waltham, USA) and run on a 0.8% agarose gel [stained with 0.5 µg/ml ethidium bromide (EtBr)] and visualised on Gel Documentation system (AlphaImager® HP, ProteinSimple, San Jose, USA). For EMSA on native PAGE, 30 µl sample was loaded per well and run at 70 V for approximately 120 minutes in a refrigerator at 2–8 °C. After the run, the gel was stained with 0.5 µg/ml EtBr in 100 mL 1X Tris-acetate-EDTA (TAE) buffer for 30 minutes and visualised on the gel documentation system. DNA-LL37 complex formation was also confirmed by confocal microscopy. DNA was labelled with Hoechst for 30 min in the dark at RT and incubated with LL37 to form complexes. Hoechst labelled DNA-LL37 complexes were treated with DNase I. After washing, samples were mounted on slides with ProLong Gold^TM^ antifade mountant (Invitrogen, Carlsbad, USA) and examined under a confocal microscope (Olympus FV10–03, software FV10i) (Olympus Corporation, Tokyo, Japan), where DNA-LL37 complexes were visible as DNase resistant aggregates.

#### Nuclease protection assay

To study whether LL37 protects DNA from serum nucleases, a nuclease protection assay was performed. Self-DNA (20 μg/ml) was incubated overnight at 37 °C in RPMI supplemented with 50% human serum (obtained from the same subject) either in the presence or absence of LL37 (100 μg/ml). The incubation was terminated by the addition of NaCl (final concentration, 1 M). The samples were then analysed by electrophoresis and visualised on a gel documentation system^43^.

### Uptake of DNA-LL37 complexes by pDCs and monocytes

#### Confocal imaging

Self-DNA was labelled with Alexa488 using the Ulysis^TM^ Nucleic Acid Labelling Kit (Invitrogen, Carlsbad, USA), as per manufacturer’s instructions. The pDCs and monocytes isolated from HC subjects were incubated with Alexa488 DNA-LL37 complexes for 60 minutes and 12 hours, respectively, at 37 °C in an incubator with 5% CO_2_. Stimulated pDCs and monocytes were then seeded and fixed by 4% paraformaldehyde (PFA) on coverslips. Cells were stained with Phycoerythrin (PE) anti–BDCA-2 antibody (clone AC144) (Miltenyi biotec, GmBh) for pDCs and PE anti-CD14 antibody (clone M5E2) (BD Biosciences) or Phalloidin-tetramethylrhodamine B isothiocyanate (TRITC) for monocytes. After washing, slides were mounted in ProLong Gold^TM^ antifade mountant with (4′,6-diamidino-2-phenylindole) DAPI (Invitrogen, Carlsbad, USA) and examined with a confocal microscope (Olympus FV10–03, software FV10i) (Olympus Corporation, Tokyo, Japan).

#### Flow cytometry

The pDCs and monocytes (at least 10^3^) isolated from HC subjects were incubated with Alexa Fluor 488-labelled DNA-LL37 complexes at different time points (pDCs: 5, 15, 30, 60 minutes; monocytes: 0.5, 1, 3, 6, 12 hours) at 37 °C in an incubator with 5% CO_2_. The uptake was terminated by adding 200 µL of 2% PFA and samples were stored at 4 °C until acquisition. Both pDCs and monocytes incubated with 2% PFA in the presence of Alexa Fluor 488 DNA-LL37 complexes served as negative controls. After incubation, cells were stained using anti-BDCA-2 PE (clone AC144) (Miltenyi Biotec GmBh) for pDCs and anti-CD14 PE (clone M5E2) (BD Biosciences, CA, USA) for monocytes and acquired on the flow cytometer. The percentage of Alexa Fluor 488 DNA-LL37 positive cells were assessed. In some experiments, pDCs and monocytes isolated from T1D and HC subjects were incubated with Alexa Fluor 488 labelled DNA-LL37 complexes and Alexa Fluor 488 labelled DNA alone for 30 minutes and 12 hours respectively and acquired by flow cytometry as described above.

### Analysis of the expression of IDO1 in monocytes

Monocytes were isolated from PBMCs of T1D and control subjects using their adherence property and removal of non-adherent cells, followed by trypsinization for detachment of monocytes. Total RNA was isolated from monocytes using TRIzol (Invitrogen), and complementary DNA (cDNA) was generated using SuperScript-III cDNA synthesis kit (Invitrogen) according to the manufacturer’s instructions. Quantitative real time-polymerase chain reaction (qRT-PCR) was performed to evaluate the expression of IDO1 and 18S (housekeeping gene) using appropriate Taqman primer probes. Data were analysed for relative quantification by the comparative ∆∆ cycle threshold (Ct) method.

### Intracellular staining for IFN-α and IDO1

After the completion of the overnight incubation period with DNA-LL37 complexes or DNA alone, both pDCs and monocytes were subjected to direct intracellular cytokine staining. The surface stained cells were fixed and permeabilized using BD Cytofix/Cytoperm™ kit (BD Biosciences) according to the manufacturer’s instructions. After permeabilization, the pDCs were stained intracellularly with anti-human IFN-α BV421 (clone 7N4–1) (BD Biosciences) IDO1 FITC (Biolegend), and monocytes with IFN-α BV421 only followed by 30-minute incubation at room temperature in dark. The cells were finally washed with 1X PBS and acquired on the flow cytometer.

### Analysis of expression of major histocompatibility complex (MHC) class II and costimulatory molecules on monocytes and pDCs

The levels of MHC class II and costimulatory molecules expressed on monocytes and pDCs were assessed by flow cytometry from their overnight culture with DNA-LL37 complexes or DNA alone, pDCs were surface stained with lineage negative cocktail (FITC labelled anti-human CD3, CD14, CD16, CD19, CD20, CD56), anti-human BDCA-2 FITC or BDCA-2 PE (clone AC144) (Miltenyi biotec GmBh), CD123 PE (clone 7G3), HLA-DR, DP, DQ BV421 (clone Tu39), Immunoglobulin-like transcript 7 (ILT7) allophycocyanin (APC) (Clone 17G10.2), CD80 PE (clone L307.4) and CD86 PE Cy7(clone 2331 FUN-1) antibodies (BD Biosciences). Similarly, monocytes were stained by anti-human CD14 Peridinin Chlorophyll Protein (PerCP) (clone MΦP9) and HLA-DR, DP, DQ BV421, CD80 and CD86 antibodies (BD Biosciences).

### *In vitro* priming of CD4 + T cells by stimulated pDCs and monocytes

pDCs (1 × 10^4^) and monocytes (1 × 10^4^) were first incubated with or without beta cell-specific antigen (preproinsulin, 10 μg/ml) (Peprotech, Israel) and with or without DNA-LL37 complexes overnight at 37 °C with 5% CO_2_ in CTS^TM^ optimizer^TM^ T cell expansion serum-free media (Thermofisher Scientific) in 96 well tissue culture plate. CD4 + T cells were then added to the culture at a ratio of 10:1 (CD4 + T cells: pDCs or monocytes) and were incubated at 37 °C with 5% CO_2_ for 4 days in presence of interleukin (IL)-2 (200 IU/mL). CD4 + T cells stimulated with anti-CD3/CD28 beads (Dyna Beads^TM^, Invitrogen) in the presence of preproinsulin were used as a positive control. After incubation, CD4 + T cells were processed for expression analysis of early activation markers CD69 and intermediate activation marker CD71. Activated CD4 + T cells were collected and labelled with anti-human CD3 BV421 (clone SK7), CD4 FITC (clone RPA-T4), CD69 PE (clone FN50), CD71 APC (clone M-A712) (each from BD Biosciences) antibodies and acquired on the flow cytometer. Representative flow cytograms of CD4 + T cell activation with controls are given in supplementary results (Supplementary Figure S4).

### Analysis of beta cell apoptosis by pDCs and monocytes

The 1.1B4 cells (beta cell line) were cultured in complete RPMI medium containing 11.1 mM glucose in the incubator at 37 °C with 5% CO_2_^44^. Transwell assay was modified and used to assess apoptosis of beta cell line^45^. Briefly, in the lower chamber of a 24-well culture plate, 1 × 10^4^ beta cells in 500 µL of complete RPMI medium were added whereas pDCs or monocytes (stimulated with DNA-LL37 complexes) were added at different concentrations in the upper chamber of the transwell insert having 0.4 μm pore size (BD Biosciences) in each well to standardize the optimal numbers of pDCs and monocytes. After standardization, a ratio of 1:20 (pDCs/monocytes: 1.1B4 cells) was used for all experiments. After 24-hours of co-culture, the beta cells were stained with Annexin V and PI (BioRad, CA, USA) according to the manufacturer’s protocol and acquired on the flow cytometer. IFN-α (2000 IU/mL) (Peprotech) was used as a positive control.

### Statistical analysis

Firstly, the data were checked for normality using the D’Agostino-Pearson Omnibus test. For normally distributed data, unpaired Student’s t-test [95% confidence interval (CI)] was used to compare continuous data i.e means between two groups, while paired Student’s t-test (95% CI) was used to compare means within the same group. Mann-Whitney U test was used for unequally distributed data. Comparisons between multiple groups were performed using a one-way analysis of variance (ANOVA) followed by Tukey’s multiple comparison test to compare all the groups. All statistical analysis was performed using GraphPad Prism 7.0 software (GraphPad Prism, La Jolla, CA). Results were expressed as mean ± standard error of mean (SEM), and p-values < 0.05 were considered statistically significant.

### Accordance

All the methods and experiments were carried out in accordance with the guidelines and regulations of IEC of PGIMER, Chandigarh, India.

### Informed consent (for experiments involving humans or human tissue samples)

We state that before taking the blood sample duly signed informed consent was obtained from all participants and/or their legal guardian/s in case of minor subjects.

## Results

### Clinical characteristics

Mean (±SEM) fasting plasma C-peptide levels were significantly lower in subjects with T1D (0.43 ± 0.05 ng/ml) than HC (2.61 ± 0.29 ng/ml) (P < 0.0001), whereas mean (±SEM) HbA1c (%) was significantly higher in T1D (11.04 ± 0.37%) as compared to HC subjects (5.21 ± 0.07%) (P < 0.0001). None of the recruited subjects had any infectious diseases or any other autoimmune disease, including celiac disease at the time of recruitment. Among the subjects with T1D, 18% of subjects were triple antibody-positive, 36% were double antibody-positive subjects (20% were anti-GAD65 and IA-2 positive, 11% were anti-GAD65, and insulin-positive and 5% were IA-2 and insulin-positive). Overall, 69% of the T1D subjects had anti-GAD65 autoantibodies, 42% had anti-IA-2 autoantibodies, and 29% had anti-insulin autoantibodies (Table 1).

### LL37 forms stable complexes with self-DNA and protects it from DNase degradation

**Figure 1:**
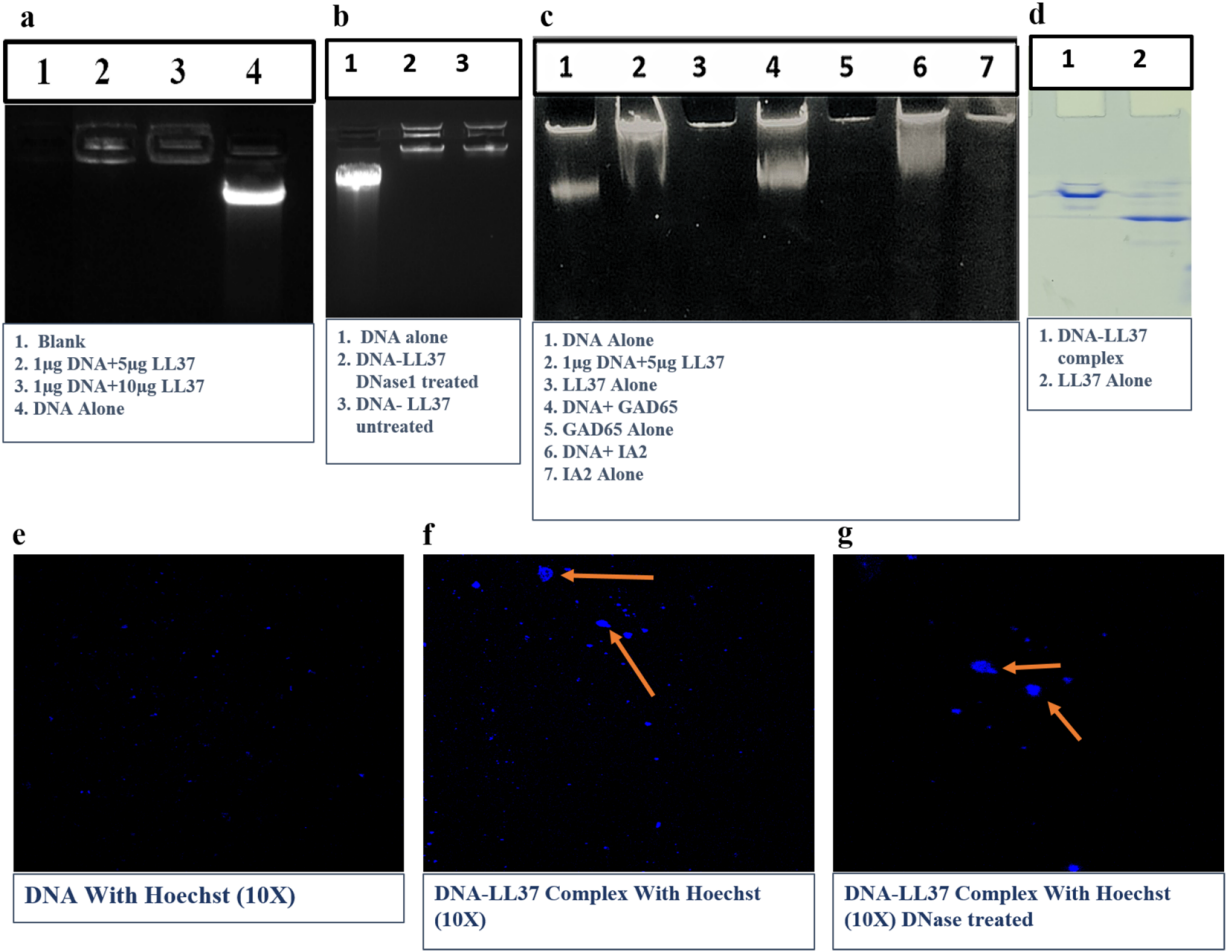
The confirmation of DNA-LL37 complexes formation by EMSA and confocal microscopy. LL37 was incubated with self-DNA at different ratios and time points. (**a**) Electrophoretic mobility shift assay (EMSA) of DNA-LL37 complexes was performed. Lanes 2 and 3 show the formation of complexes. (**b**) DNA alone (control, Lane 1), DNA complex treated with DNase 1 (lane 2) and untreated complex (lane 3). (**c**) EMSA on native PAGE gel shows the stable formation of self-DNA complexes with LL37 (lane 2), whereas self-DNA did not form complexes with other beta cell associated proteins, such as GAD65 (lane 4) and IA-2 (lane 6), (**d**) PAGE gel shows the formation of DNA-LL37 complexes (lane 1) and LL37 alone (Lane 2), (**e**) Confocal image of Hoechst labelled DNA alone and (**f**) Hoechst labelled DNA-LL37 complex, forming visible aggregates, confirming complex formation. (**g**) DNA-LL37 complexes were treated with DNase I to remove extra-unbound DNA. The exposure time was same in all procedures and images. The gel images are of single gel and do not contain cropped or cut-outs from other gels. Gels (**a, b and d**) are cropped from their respective gels to increase the clarity and conciseness to include only relevant lanes (full gel images are provided in supplementary figures S1, S2 and S3).

LL37 peptide is known to form complexes by condensing the DNA regardless of its nucleotide sequences, depending on its charge^42^. The DNA-LL37 complex formation was assessed by EMSA on a 0.8% agarose gel and native PAGE (Fig. 1a-d) and confirmed by confocal microscopy (Fig. 1e-g). The stability of the complex generation was observed at ratios of 10:1 and 5:1 (LL37: DNA) with an optimal incubation time of 30 minutes as indicated by retention of large-sized complexes in the loading wells (Fig. 1a, lane 2). The ratio of 5:1 was selected as optimal and used for all experiments at a concentration of 3 µg/100 µL in the RPMI medium. Self-DNA in both intracellular and extracellular environments does not act as alarmin because of its rapid degradation by DNases; hence we interrogated whether binding of LL37 peptide confers any protection to the DNA. The DNA-LL37 complex was treated with DNase I (Fig. 1b, lane 2) and was run on agarose gel along with untreated DNA-LL37 complex (Fig. 1b, lane 3). As expected, no difference was observed between the treated and untreated DNA-LL37 complexes, confirming protection from DNase degradation. In the agarose gel, DNA-LL37 complexes did not appear as bright as DNA alone, as LL37 interferes with the intercalating dyes like EtBr. Therefore, we performed EMSA on a PAGE gel, by incubating DNA with EtBr before generating complexes with LL37. The assay was sensitive enough to determine the formation of stable DNA-LL37 complexes (Fig. 1c, lane 2), whereas self-DNA did not form complexes with other proteins, such as GAD65 (Fig. 1c, lane 4) and IA-2 (Fig. 1c, lane 6) which were used as negative controls. We also confirmed the formation of DNA-LL37 complexes on PAGE gel, where DNA-LL37 complexes were retained at the intersection of stacking and resolving gel (Fig. 1d lane 1), whereas LL37 moved further due to its smaller size (Fig. 1d lane 2).

Next, the DNA-LL37 complex formation was confirmed by confocal microscopy in which we could demonstrate that LL37 binds to Hoechst labelled DNA to form visible aggregates (Fig. 1f) that were resistant to DNase treatment (Fig. 1g) as compared to Hoechst labelled DNA alone (Fig. 1e). Collectively, we were able to demonstrate the formation of DNA-LL37 complexes that protected self-DNA from DNase degradation.

### Binding of LL37 efficiently transports self-DNA to the cytoplasm of monocytes and pDCs

**Figure 2:**
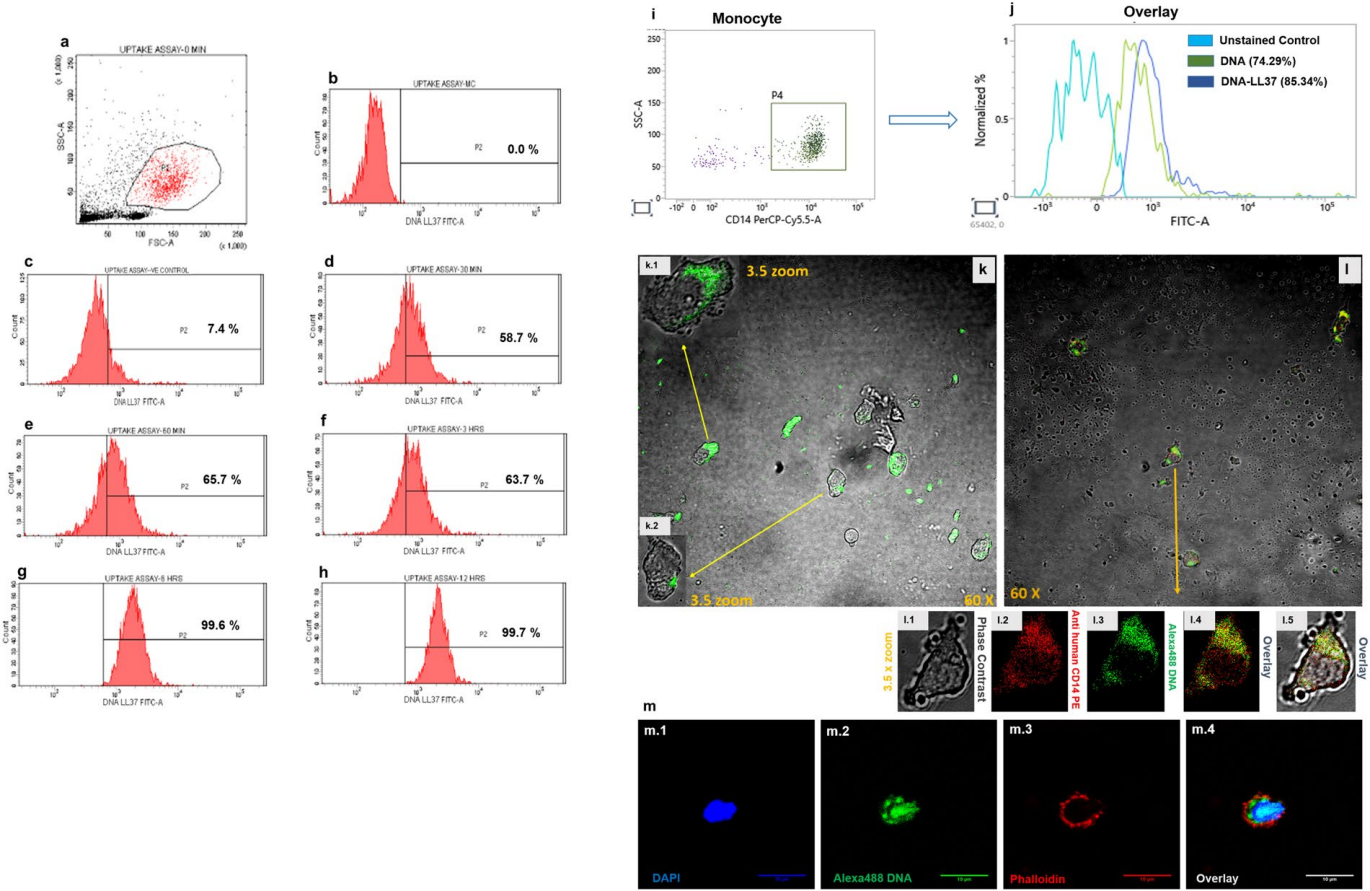
The uptake of DNA-LL37 complexes by monocytes. (**a-h**) Representative flow cytograms showing the internalization of Alexa488 DNA-LL37 complexes by monocytes isolated from healthy controls at different time points (0.5, 1, 3, 6 and 12 hours). (**a**) Gating of monocytes by FSC and SSC, (**b**) Unstimulated cells, (**c**) negative control (PFA treated cells) showing minimal uptake (7.4 ± 0.29%), (**d**) uptake at 0.5 hours (58.7 ± 0.95%), (**e**) uptake at 1 hour (65.7 ± 0.67%), (**f**) uptake at 3 hours (63.7 ± 1.16%), (**g**) uptake at 6 hours (99.6 ± 0.2%), (**h**) uptake at 12 hours (99.7 ± 0.1%). Monocytes became saturated with complexes after 6 hours of treatment. Data is presented as mean ± SEM from 3 independent experiments. (**i,j**) Representative flow cytograms showing the internalization of Alexa488 DNA alone and Alexa488 DNA-LL37 complexes by monocytes isolated from healthy controls. (**i**) SSC and CD14 were used to gate monocytes, (**j**) uptake of Alexa488 DNA alone (74.29 ± 0.005%) was less than the uptake of Alexa488 DNA-LL37 complexes (85.34 ± 0.005%) (p = 0.001). Data is presented as mean ± SEM from 3 independent experiments. (**k-m**) Representative confocal images showing uptake of Alexa488 labelled DNA-LL37 complexes by monocytes of healthy controls. (**k**) Monocytes showed cytoplasmic localization of Alexa488 DNA-LL37 complexes (green). (**l and m**) The cytoplasmic uptake was confirmed by merged overlay images, l.1) Phase contrast, l.2) Cell membrane marked by CD14 (red), l.3) Alexa488 DNA-LL37 complexes (green) clearly showing the cytoplasmic location of complexes in overlay images (l.4 and l.5). m.1) Monocyte nucleus stained with DAPI, m.2) Alexa488 DNA-LL37 complexes (green), m.3) Cell membrane marked by Phalloidin dye (red), m.4) Overlay images showing the cytoplasmic location of complexes. All images are at 60X magnification with 3.5X zoom on selected cells.

**Figure 3:**
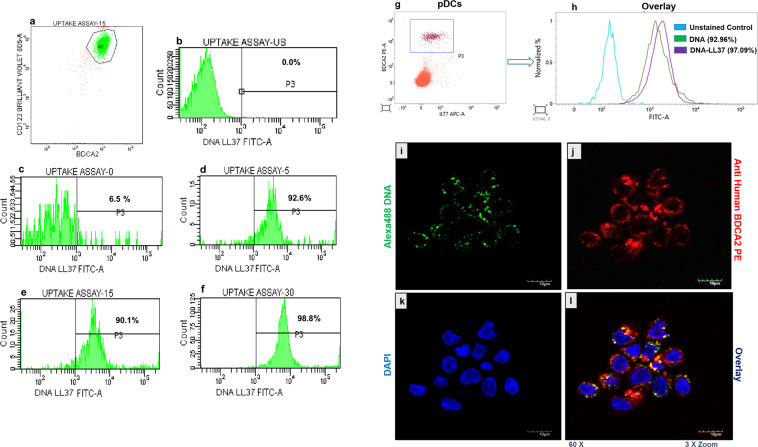
The uptake of DNA-LL37 complexes by pDCs. (**a-f**) Representative flow cytograms showing the internalization of Alexa488 DNA-LL37 complexes by pDCs isolated from healthy controls at different time points (5, 15 and 30 minutes). (**a**) CD123 + BDCA-2+ pDCs, (**b**) Unstimulated cells, (**c**) Negative control (PFA treated cells) showing minimal uptake (6.5 ± 0.12%), (**d**) Uptake at 5 minutes (92.6 ± 0.85%), (**e**) Uptake at 15 minutes (90.1 ± 0.61%), (**f**) Uptake at 30 minutes (98.8 ± 0.52%). Uptake of complexes by pDCs occurred at a very fast rate as shown by their saturation just in 30 minutes of treatment with the complexes. Data is presented as mean ± SEM from 3 independent experiments. (**g,h**) Representative flow cytograms showing the internalization of Alexa488 DNA alone and Alexa488 DNA-LL37 complexes by pDCs of healthy controls. (**g**) BDCA-2 PE and ILT7 were used to gate pDCs, (**h**) uptake of Alexa488 DNA alone (92.96 ± 0.87%) and the uptake of Alexa488 DNA-LL37 complexes (97.09 ± 0.86%) (p = 0.01). Data is presented as mean+SEM from 3 independent experiments. (**i-l**) The representative confocal images showing uptake of Alexa488 DNA-LL37 complexes by pDCs of healthy controls. (**i**) Alexa488 DNA-LL37 complex (green), (**j**) Cell membrane marked by BDCA-2 PE (red) and (**k**) Nucleus (blue). (**l**) Merged overlay images showing the cytoplasmic location of complexes. All images were acquired at 60X magnification with 3X zoom on selected cells with a bar representing 10 µm on the scale.

We assessed the time required for the complete uptake of DNA-LL37 complex by incubating monocytes with Alexa Fluor 488 labelled DNA-LL37 complexes at different time points (Fig. 2a–h). Monocytes internalized DNA-LL37 complexes immediately, with 58.7 ± 0.95% of monocytes showing internalized complexes (Fig. 2d) at 30 minutes with saturation at 6 hours of treatment (Fig. 2g), that was stable even after 12 hours (Fig. 2h). Hence, 12 hours was selected as the optimal incubation time for stimulation experiments. DNA-LL37 complexes were also efficiently internalized by pDCs (Fig. 3a–f), immediately after treatment with 92.6 ± 0.85% of pDCs showing internalized complexes within 5 minutes (Fig. 3d) and saturation at 30 minutes (Fig. 3f).

To further compare differential uptake of DNA-LL37 complexes vs DNA alone, monocytes and pDCs isolated from representative HC and T1D subjects were incubated with DNA-LL37 complexes and DNA alone for 12 hours and 30 minutes respectively and frequency of monocytes and pDCs in the two settings were compared. We observed an increase in the frequency of monocytes taking up DNA-LL37 complexes (85.34 ± 0.005%) than DNA alone (74.29 ± 0.005%) (p = 0.001) (Fig. 2i,j). Similarly, a significant increase in the frequency of pDCs taking up DNA-LL37 complexes (97.09 ± 0.86%) was observed than DNA alone (92.96 ± 0.87%) (p = 0.01) (Fig. 3g,h). Additionally, to determine the quantity of uptake of DNA-LL37 complex on per cell basis, we also analysed the mean fluorescent intensity (MFI) and observed increased uptake when DNA formed complexes with LL37 versus DNA alone (MFI, 3219 ± 343 vs 1308 ± 95) (p = 0.02) in monocytes and (MFI, 7039 ± 356 vs 4994 ± 193) (p = 0.01) in pDCs, respectively. DNA alone was detected in the pDCs and monocytes because we had not used any nuclease during the experiment, to deliberately allow DNA to enter pDCs and monocytes for the sake of comparison. To confirm that binding of LL37 enhances uptake of DNA in a natural extracellular environment, we added autologous serum (50% in culture media) to the monocytes and pDCs incubated with DNA alone and observed significantly lower uptake of DNA alone (p = 0.001) in cells incubated with serum in comparison to cells incubated with DNA alone in a nuclease-free medium (Supplementary Figure S5c). Additionally, we also compared the uptake of DNA-LL37 complexes in pDCs and monocytes from T1D and HC subjects and observed no difference in the uptake of DNA-LL37 complexes between the pDCs or monocytes of the two groups (Supplementary Figure S5a,b).

Finally, we assessed the uptake of labelled DNA-LL37 complexes in the monocytes and pDCs separately and observed their internalization by confocal microscopy. Here, we were able to clearly demonstrate intracellular localization of the DNA-LL37 complexes in the cytoplasm of monocytes (Fig. 2k-m) and pDCs (Fig. 3i-l). The distribution of DNA-LL37 complexes appeared to be diffused in the cytoplasm of monocytes, giving an impression of nuclear localization, however, upon examining the magnified images (Fig. 2k.1, k.2) and overlay images (Fig. 2, l.4 and l.5), the cytoplasmic distribution of DNA-LL37 complexes was confirmed.

### Monocytes and pDCs of subjects with T1D show increased expression of IFN-α following stimulation with DNA-LL37 complexes

**Figure 4:**
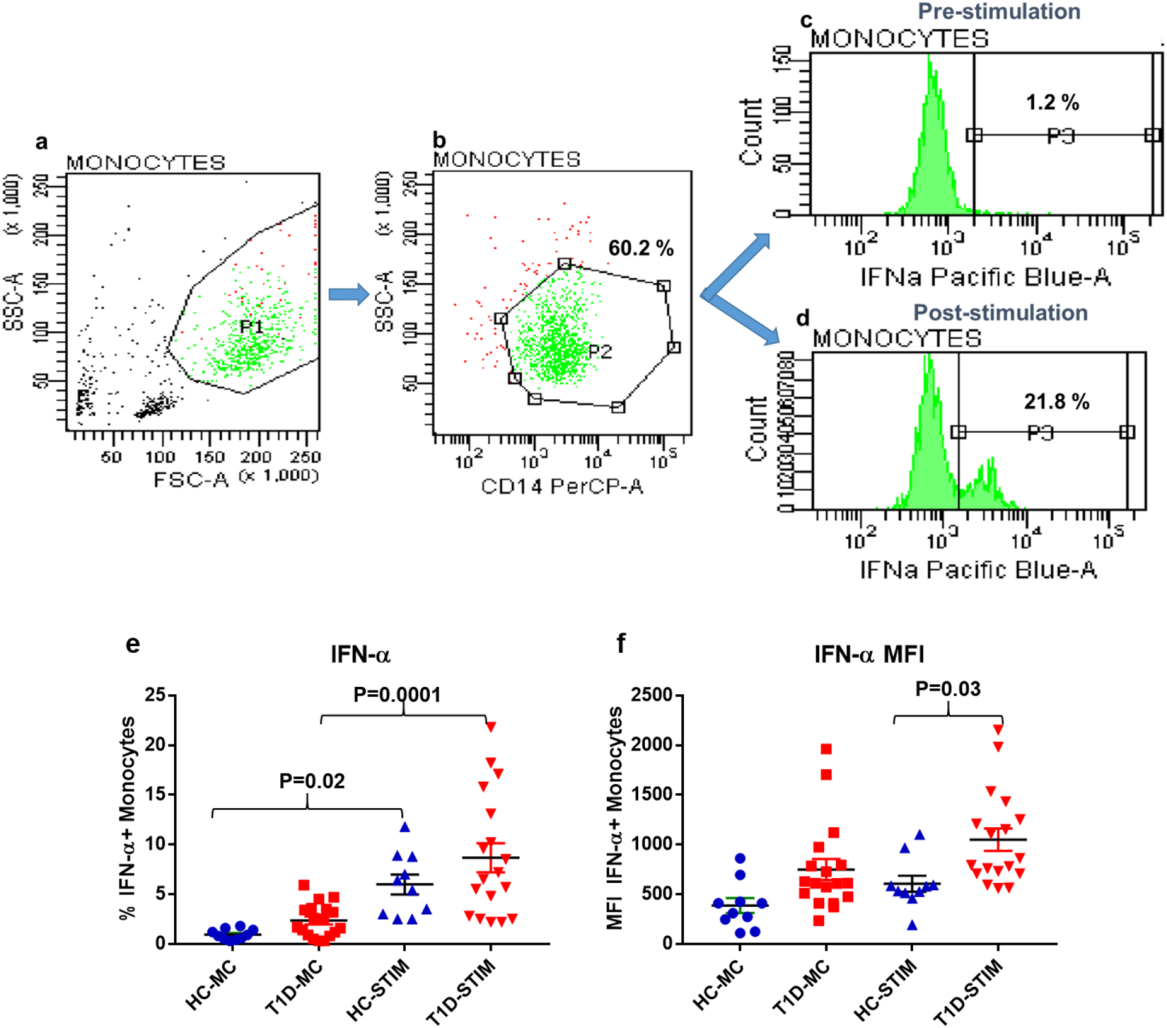
The effect of DNA-LL37 complexes stimulation on the expression of IFN-α by monocytes of T1D and HC subjects. Representative flow cytograms showing the expression of intracellular IFN-α by monocytes. (**a**) Gating of monocytes based on FSC and SSC, (**b**) CD14 + monocytes, (**c, d**) Intracellular expression of IFN-α prior to after stimulation with DNA-LL37 complexes. (**e**) Frequency of IFN-α producing monocytes in T1D (n = 18) and HC (n = 10) subjects both prior to (MC) and after stimulation (Stim) with DNA-LL37 complexes (3 µg complex per 100 µL of media). (**f**) MFI of IFN-α producing monocytes in T1D and HC subjects before and after stimulation with DNA-LL37 complexes. Data is presented as mean (±SEM) frequency and one-way ANOVA followed by Tukey’s multiple comparison test was used to compare the means. P  <  0.05 was considered statistically significant. Relevant FMO tubes were used to set gates.

**Figure 5:**
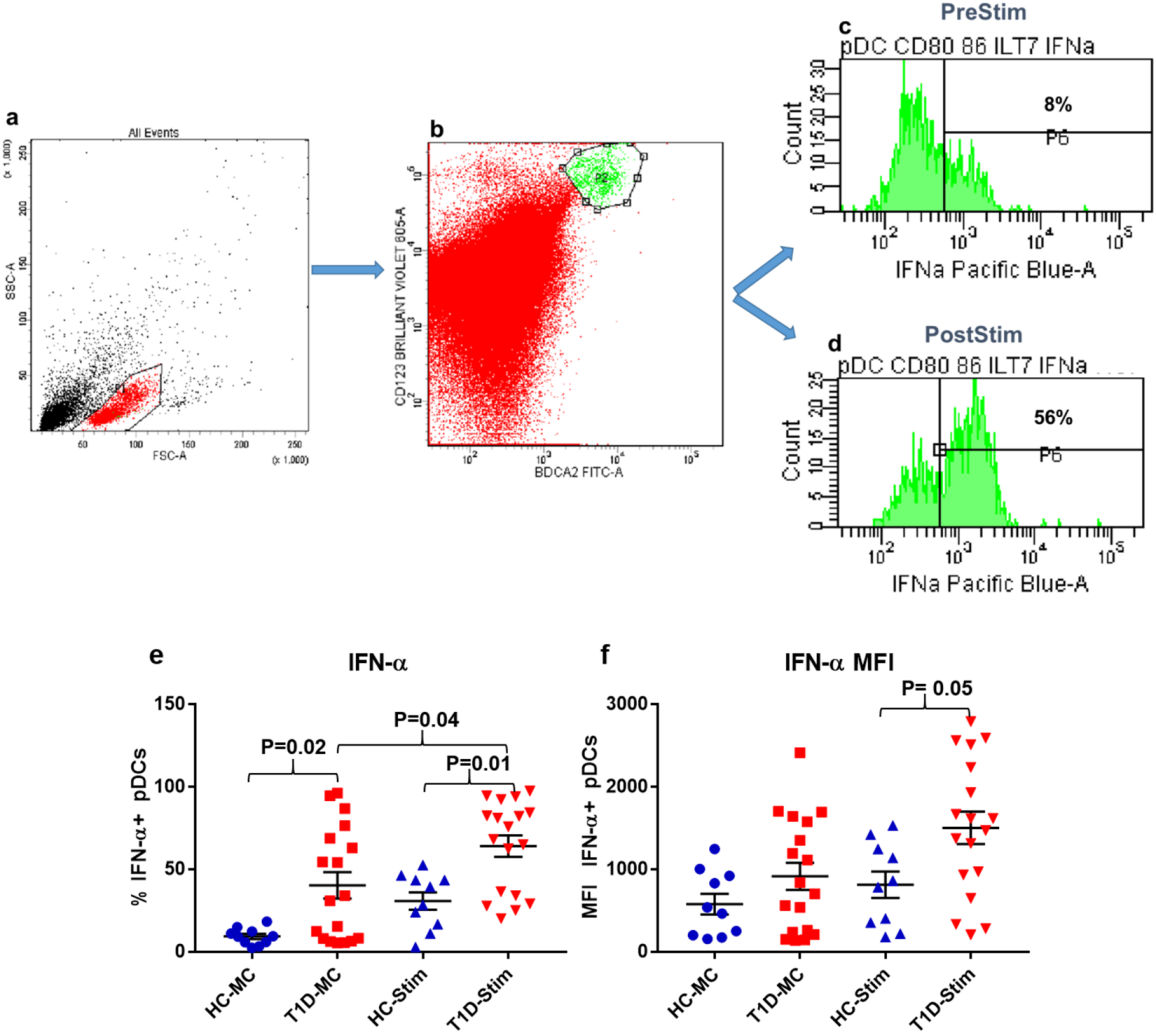
The effect of DNA-LL37 complexes stimulation on the expression of IFN-α by pDCs of T1D and HC subjects. Representative flow cytograms showing the expression of intracellular IFN-α by pDCs, prior to after stimulation with DNA-LL37 complexes. (**a**) Gating of lymphocytes (**b**) Gating of pDCs. (**c, d**) Intracellular expression of IFN-α by pDCs before and after stimulation with DNA-LL37 complexes. (**e**) Frequency of IFN-α producing pDCs in T1D (n = 18) and HC (n = 10) subjects both prior to (MC) and after stimulation (Stim) with DNA-LL37 complexes (3 µg complex per 100 µL of media). (**f**) MFI of IFN-α producing pDCs in T1D and HC subjects both before and after stimulation with DNA-LL37 complexes. Data is presented as mean (±SEM) frequency and one-way ANOVA followed by Tukey’s multiple comparison test was used to compare the means. P  <  0.05 was considered statistically significant. Relevant FMO tubes were used to set gates.

Firstly, we stimulated pDCs and monocytes with DNA alone and DNA-LL37 complexes to compare the effects of their uptake on all subjects. After uptake of DNA alone, the pDCs and monocytes expressed significantly lower levels of IFN-α in comparison to DNA-LL37 complexes (p = 0.02) and (p = 0.03) (Supplementary Figure S6 and S7). Next, we stimulated monocytes and pDCs with DNA-LL37 complexes to see whether they could activate their cellular machinery required for the expression of IFN-α (Figs. 4a-d and 5a-d). Overall, we observed an increased frequency of cells expressing IFN-α after stimulation of monocytes from both T1D (p = 0.0001) and HC subjects (p = 0.02) (Fig. 4e) and stimulation of pDCs from T1D subjects (p = 0.04) (Fig. 5e) in comparison to unstimulated controls. Variance in the frequency of monocytes and pDCs expressing IFN-α, prior to and after stimulation, among the subject groups was compared. The monocytes in the peripheral circulation of both T1D and HC subjects had a lower and comparable frequency of IFN-α + monocytes in the absence of any stimulation (2.3 ± 0.39% vs 0.95 ± 0.17%) (Fig. 4e). Similarly, the monocytes of T1D and healthy controls also showed comparable frequency of IFN-α + monocytes following stimulation with DNA-LL37 complexes (8.7 ± 1.5% vs 6.0 ± 1.0%) (Fig. 4e). On analysis of MFI data, we observed that monocytes of the T1D group demonstrated higher expression of IFN-α following stimulation with DNA-LL37 complexes (1049 ± 112 vs 605 ± 81) (p = 0.03) (Fig. 4f) in comparison to HC group.

In contrast to monocytes, the pDCs in the peripheral blood circulation of T1D group showed a significantly higher frequency of pDCs expressing IFN-α than HC subjects (40.48 ± 8.0% vs 9.38 ± 1.6% in unstimulated), indicating that the pDCs are in an activated state in the subjects with T1D (p = 0.02) (Fig. 5e). Higher frequency of IFN-α + pDCs was also observed after stimulation of pDCs from T1D subjects in comparison to HC subjects (64.16 ± 6.50% vs 30.94 ± 5.3%) (p = 0.01) (Fig. 5e). The MFI data as well suggested higher expression of IFN-α in the T1D group after stimulation with DNA-LL37 complexes (1505 ± 195 vs 816 ± 160) (p = 0.05) (Fig. 5f) in comparison to HC group.

### DNA-LL37 complexes increase the antigen presentation capacity of pDCs and monocytes

**Figure 6:**
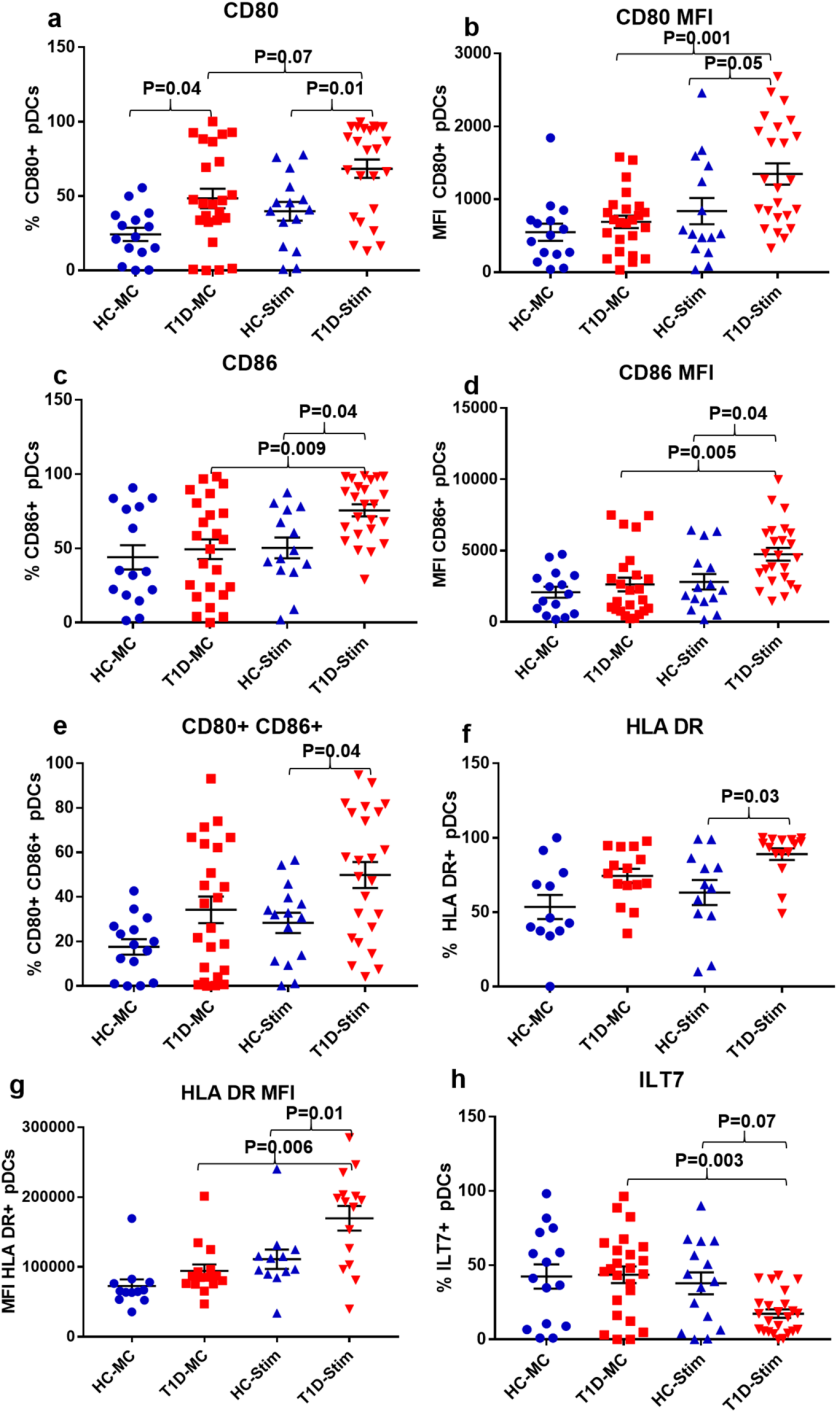
The effect of DNA-LL37 complexes stimulation on the antigen presentation capacity of pDCs of T1D and HC subjects. Following stimulation with DNA-LL37 complex (3 µg complex per 100 µL of media), pDCs were assessed for the expression of markers of antigen presentation (HLA-DR) and costimulation (CD80, CD86) and compared to unstimulated pDCs (MC) within the groups and between the T1D (n = 24) and HC (n = 15) groups. (**a**) Frequency of CD80 + pDCs, (**b**) MFI of CD80 on pDCs, (**c**) Frequency of CD86 + pDCs, (**d**) MFI of CD86 on pDCs, (**e**) Frequency of pDCs co-expressing CD80 and CD86, (**f**) Frequency of ILT7 expressing pDCs, (**g**) Frequency of HLA-DR expressing pDCs and (**h**) MFI of HLA-DR on pDCs. The number of subjects analysed for HLA-DR expression were; T1D (n = 15) and HC (n = 12). Data is presented as mean (±SEM) frequency or MFI, and one-way ANOVA followed by Tukey’s multiple comparison test was used to compare the means. P  <  0.05 was considered statistically significant. Relevant FMO tubes were used to set gates.

Here, at first, we stimulated the pDCs and monocytes with DNA alone and DNA-LL37 complexes to compare the effect of their uptake in all subjects. After uptake of DNA alone, we observed a significantly lower frequency of pDCs expressing CD80 (p = 0.01), CD86 (p = 0.008) and HLA-DR (p = 0.02) (Supplementary Figures S6) as compared to uptake of DNA-LL37 complexes. A similar trend was observed for monocytes too, with a significantly lower frequency of monocytes expressing CD80 (p = 0.005), CD86 (p = 0.02) and HLA-DR (p = 0.003) (Supplementary Figure S7). Next, we investigated whether uptake of DNA-LL37 complexes influences the co-stimulatory and antigen presentation capacity of pDCs and monocytes in T1D and HC groups. Post stimulation, the T1D subjects showed a significantly higher frequency of pDCs expressing CD80 (68.33 ± 6.19% vs 39.77 ± 6.3%) (p = 0.01), CD86 (75.62 ± 4.06% vs 50.26 ± 7.0%) (p = 0.04) and co-expressing CD80 and CD86 (49.9 ± 5.9% vs 28.4 ± 4.6%) (p = 0.04) than their HC counterparts (Fig. 6 a,c,e). Next, when we assessed the MFI of CD80 and CD86 on the pDCs; again the pDCs in T1D group exhibited higher MFI than the HC group, following stimulation with DNA-LL37 complexes (CD80: 1348 ± 146 vs 839 ± 180) (p = 0.05) and (CD86: 4741 ± 452 vs 2812 ± 540) (p = 0.04) (Fig. 6b,d). As an indicator of increased antigen presentation capacity, the frequency of pDCs expressing HLA-DR following stimulation with DNA-LL37 complexes was also higher in the T1D group as compared to the HC group (89.04 ± 3.9% vs 63.2 ± 8.4%) (p = 0.03) (Fig. 6f). Upon evaluation of MFI of HLA-DR on the pDCs following stimulation, we again observed a significant increase in the expression of HLA-DR in the T1D group (169744 ± 17562 vs 111024 ± 13809) (p = 0.01) (Fig. 6g).

Further, we assessed the frequency of pDCs expressing ILT7 which is downregulated following activation and maturation of pDCs^46,47^. Post DNA-LL37 complex stimulation, the frequency of pDCs expressing ILT7 was lower in T1D as compared to the HC group, but was statistically non-significant (17.37 ± 2.8% vs 37.81 ± 7.4%), (p = 0.07) (Fig. 6h).

**Figure 7:**
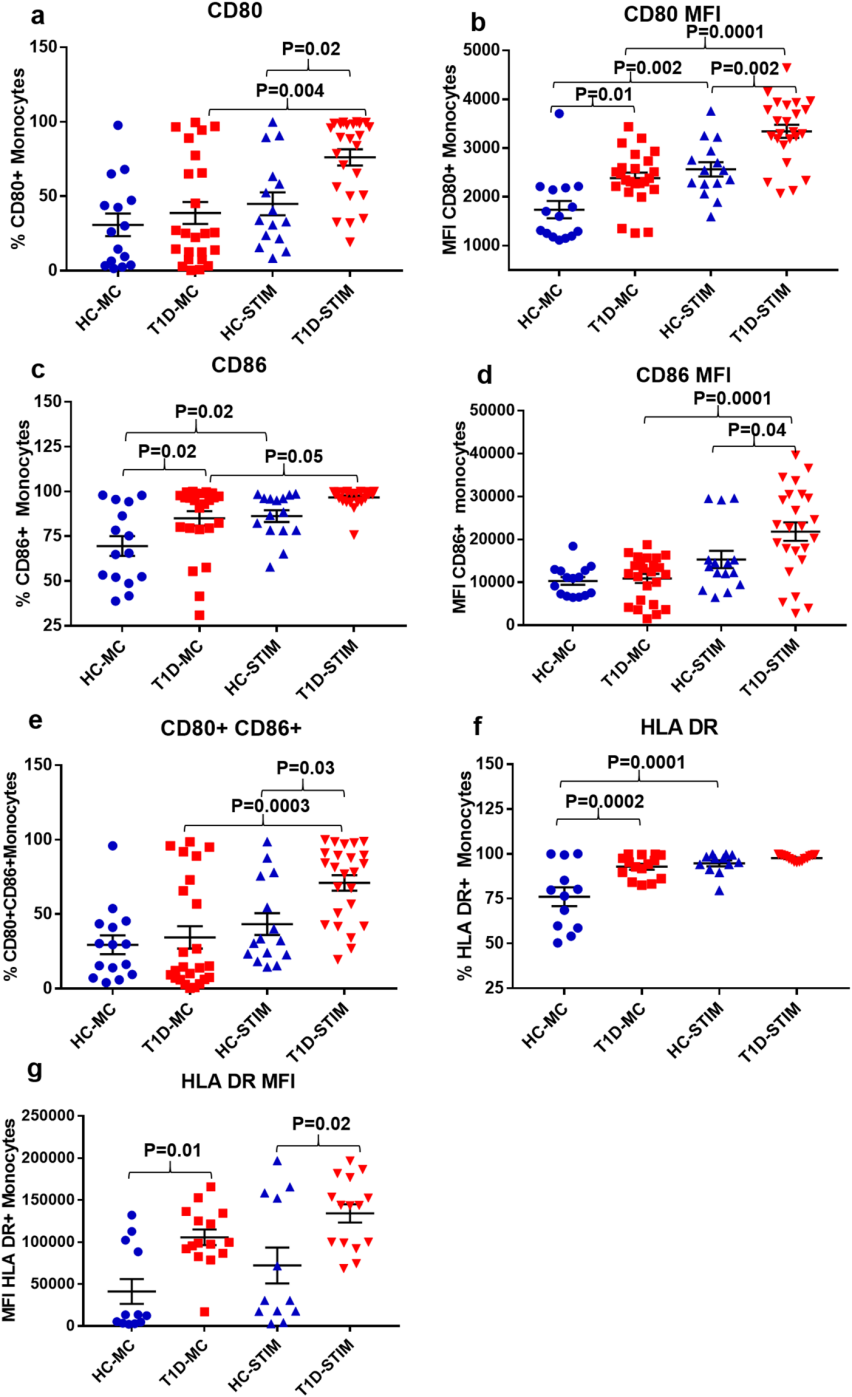
The effect of DNA-LL37 complexes stimulation on the antigen presentation capacity of monocytes of T1D and HC subjects. Following stimulation with DNA-LL37 complex (3 µg complex per 100 µL of media), monocytes were assessed for the expression of markers of antigen presentation (HLA-DR) and costimulation (CD80, CD86) and compared to unstimulated monocytes (MC) within the groups and between the T1D (n = 24) and HC (n = 15) groups. (**a**) Frequency of CD80 + monocytes, (**b**) MFI of CD80 on monocytes, (**c**) Frequency of CD86 + monocytes, (**d**) MFI of CD86 on monocytes, (**e**) Frequency of monocytes co-expressing CD80 and CD86, (**f**) Frequency of HLA-DR expressing monocytes and (**h**) MFI of HLA-DR on monocytes. The number of subjects analysed for HLA-DR expression were; T1D (n = 15) and HC (n = 12). Data is presented as mean (±SEM) frequency or MFI, and one-way ANOVA followed by Tukey’s multiple comparison test was used to compare the means. P  <  0.05 was considered statistically significant. Relevant FMO tubes were used to set gates.

Post stimulation, the T1D group displayed a higher frequency of monocytes expressing CD80 (76.16 ± 5.42% vs 45.0 ± 7.7%) (p = 0.02) but a similar frequency of CD86 + monocytes (96.68 ± 1.05% vs 86.29 ± 3.3%) as compared to the HC group, respectively. The frequency of monocytes co-expressing CD80, CD86 was also significantly higher in T1D as compared to the HC group (71.02 ± 5.2% vs 43.4 ± 7.3%) (p = 0.03) (Fig. 7 a,c,e). Additionally, when the MFI of CD80 and CD86 was compared, monocytes in T1D group exhibited higher MFI than the HC group, following stimulation with DNA-LL37 complexes (CD80: 3344 ± 135 vs 2564 ± 146) (p = 0.002) and (CD86: 21829 ± 2135 vs 15324 ± 2015) (p = 0.04) (Fig. 7b,d). To assess the antigen presenting capacity, we also evaluated the frequencies of monocytes expressing HLA-DR in both peripheral blood and following stimulation with DNA-LL37 complexes. We observed a higher frequency of peripheral monocytes expressing HLA-DR in T1D as compared to the HC group (92.83 ± 1.6% vs 76.07 ± 5.2%) (p = 0.0002) (Fig. 7f). The MFI of HLA-DR on monocytes was also significantly higher in T1D group both before and after stimulation with DNA-LL37 complexes (105588 ± 9298 vs 41098 ± 4749) (p = 0.01) and (134106 ± 10890 vs 72113 ± 1300) (p = 0.02), respectively (Fig. 7g).

### Monocytes and pDCs loaded with DNA-LL37 complexes activate CD4 + T cells

**Figure 8:**
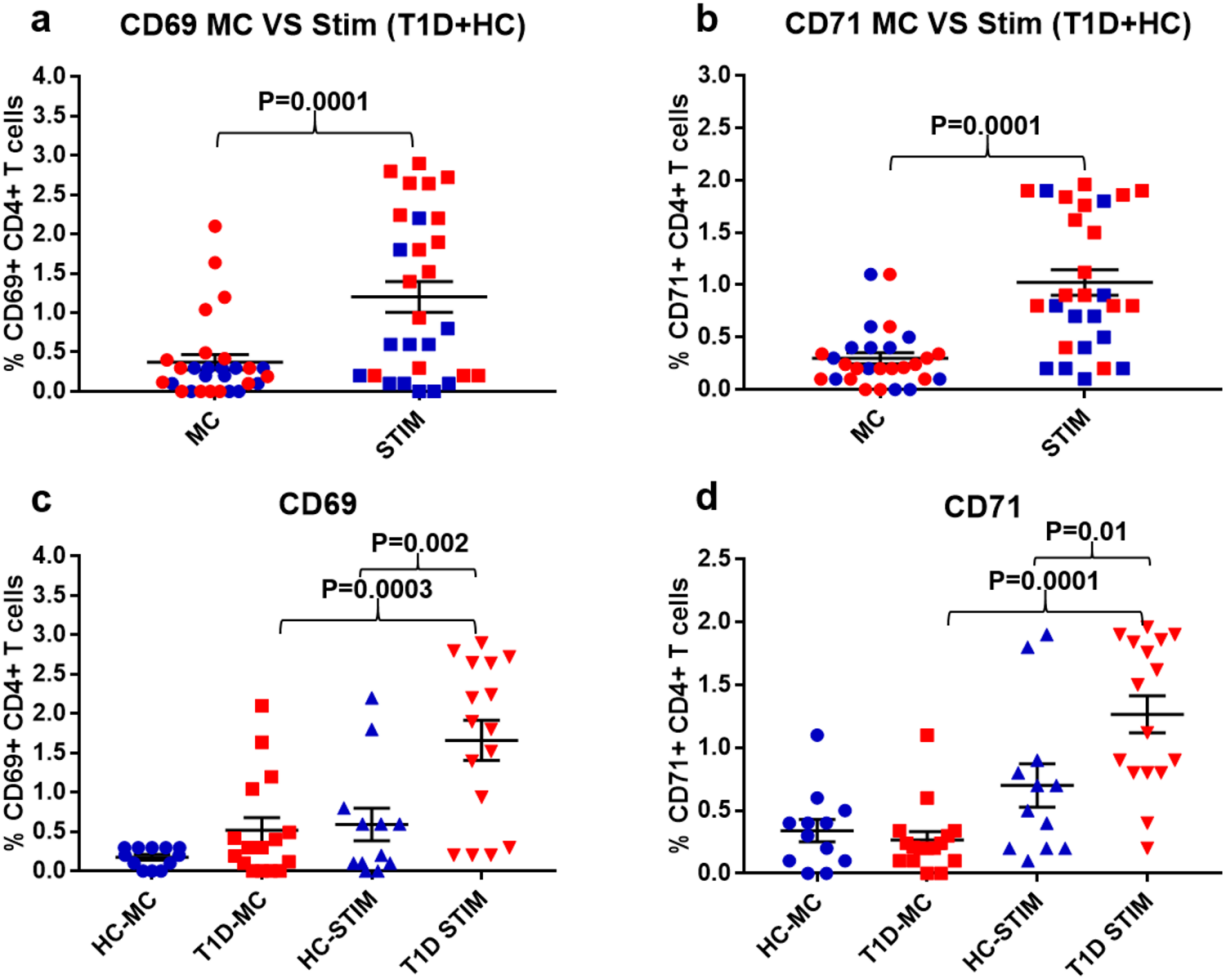
The activation of CD4 + T cells co-cultured with autologous monocytes stimulated with DNA-LL37 complexes. (**a**) Following the co-culture of CD4 + T cells with DNA-LL37 complex stimulated (Stim) and unstimulated (MC) autologous monocytes (3 µg complex per 100 µL of media), we assessed the expression of, (**a**) CD69 and (**b**) CD71 on CD4 + T cells of all subjects. (**c, d**) Activation status of CD4 + T cells in the T1D (n = 16) and HC (n = 12) subject groups was assessed by determining the frequency of CD4 + T cells expressing CD69 and CD71. Data is presented as mean ± SEM and Student’s t test was used to compare the means in figures a and b whereas, one-way ANOVA followed by Tukey’s multiple comparison test was used to compare the means in figures c and d. P  <  0.05 was considered statistically significant. Relevant FMO tubes were used to set gates.

**Figure 9:**
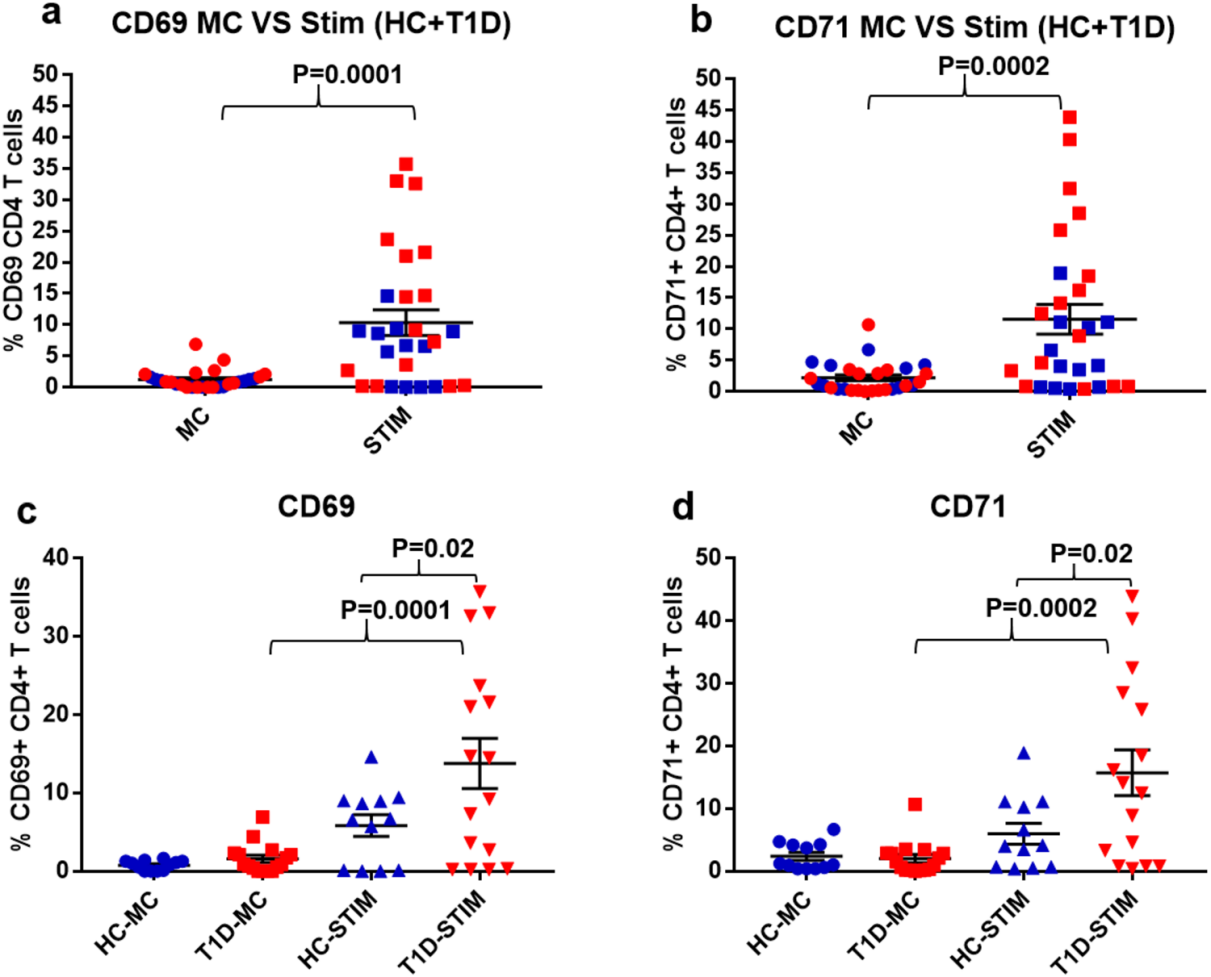
The activation of CD4 + T cells co-cultured with autologous pDCs stimulated with DNA-LL37 complexes. (**a**) Following the co-culture of CD4 + T cells with DNA-LL37 complex stimulated (Stim) and unstimulated (MC) autologous pDCs (3 µg complex per 100 µL of media), we assessed the expression of, (**a**) CD69 and b) CD71 on CD4 + T cells of all subjects. (**c, d**) Activation status of CD4 + T cells in the T1D (n = 16) and HC (n = 12) subject groups was assessed by determining the frequency of CD4 + T cells expressing CD69 and CD71. Data is presented as mean ± SEM and Student’s t test was used to compare the means in figures a and b whereas, one-way ANOVA followed by Tukey’s multiple comparison test was used to compare the means in figures c and d. P  <  0.05 was considered statistically significant. Relevant FMO tubes were used to set gates.

To evaluate whether monocytes and pDCs stimulated with DNA-LL37 complexes further activate co-cultured CD4 + T cells, we assessed the expression of activation markers, CD69 and CD71 on CD4 + T cells. We observed that, stimulated monocytes were able to activate CD4 + T cells from majority of subjects by significantly increasing the frequency of CD69 + and CD71 + CD4 + T cells in co-cultures of stimulated vs unstimulated monocytes (1.2 ± 0.19% vs 0.37 ± 0.09%) (p = 0.0001) and (1.02 ± 0.12% vs 0.29 ± 0.05%) (p = 0.0001) (Fig. 8a,b), respectively. Similarly, we observed that, stimulated pDCs were able to activate CD4 + T cells from a majority of subjects as indicated by heightened frequency of CD69 + and CD71 + CD4 + T cells in co-culture with stimulated vs unstimulated pDCs (10.72 ± 2.2% vs 1.24 ± 0.28%) (p = 0.0001) and (11.56 ± 2.3% vs 2.17 ± 0.45%) (p = 0.0002), respectively (Fig. 9a,b). Upon assessing the correlation between upregulation of CD69 + and CD71 + on CD4 + T cells, we observed a positive correlation between both the activation markers following stimulation of CD4 + T cells with pDCs (r = 0.77, p = 0.001) as well as monocytes (r = 0.48, p = 0.03) (data not shown).

Next, the effect of DNA-LL37 stimulated monocytes on CD4 + T cells isolated from T1D and HC groups was analysed separately. The frequency of CD4 + T cells expressing CD69 and CD71 was found to be significantly higher in T1D as compared to HC group (1.67 ± 0.25% vs 0.59 ± 0.2%) (p = 0.002) and (1.27 ± 0.14% vs 0.7 ± 0.17%) (p = 0.01) (Fig. 8c,d), respectively. Similarly, after co-culture with stimulated pDCs, we observed significantly higher frequency of CD4 + T cells expressing CD69 and CD71 in T1D versus HC group (13.78 ± 3.1% vs 5.8 ± 1.3%) (p = 0.02) and (15.74 ± 3.6% vs 5.9 ± 1.6%) (p = 0.02), respectively (Fig. 9c,d).

### PDCs and monocytes stimulated with DNA-LL37 complexes induce apoptosis of beta cells

To assess whether DNA-LL37 complex stimulation of monocytes and pDCs (isolated from T1D and HC subjects) had the potential to cause apoptosis of beta cells, we performed a modified transwell *in vitro* assay^45^.

**Figure 10:**
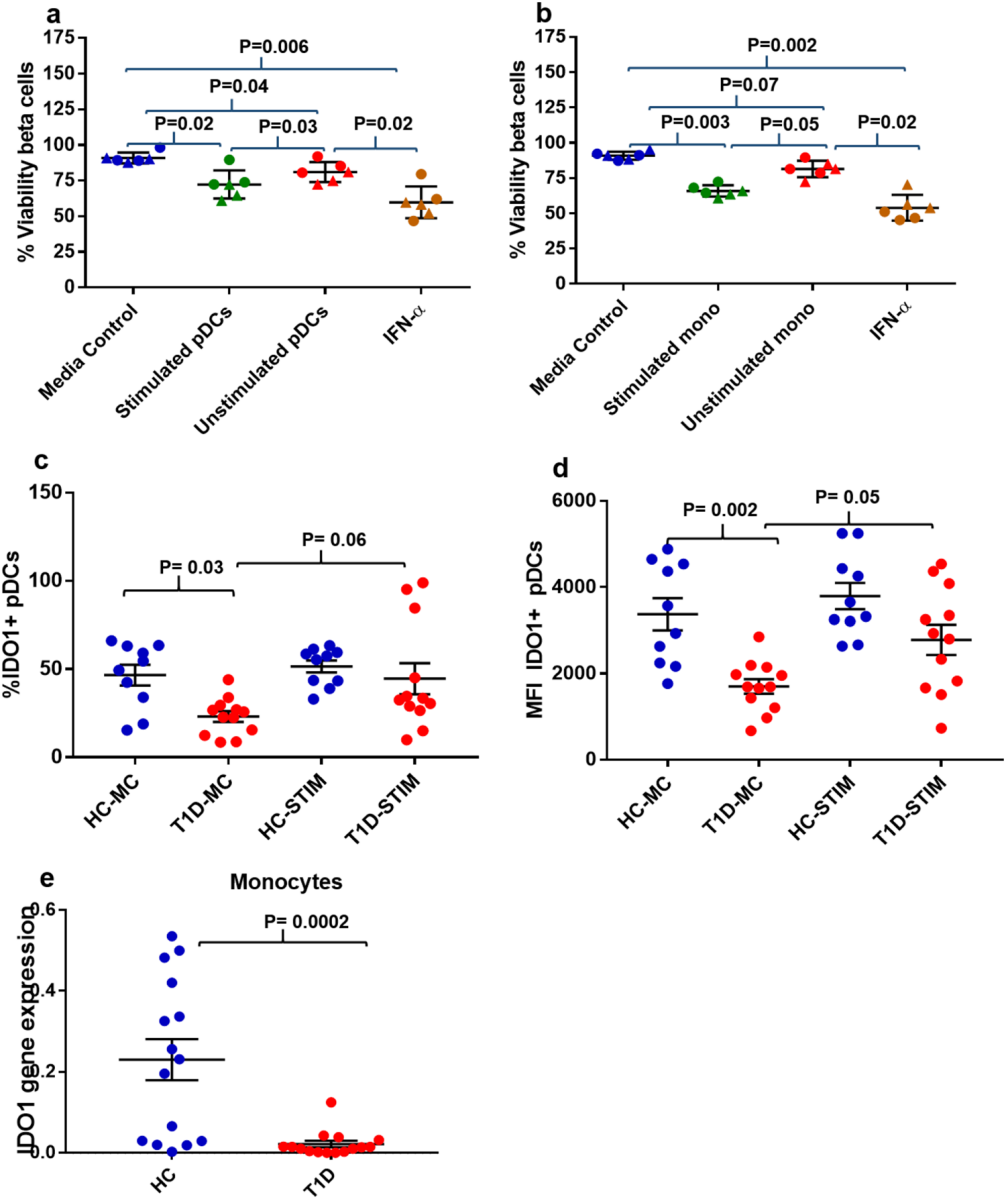
The percent viability of beta cells co-cultured with stimulated pDCs or monocytes and the expression of IDO1 in pDCs and IDO1 in monocytes. (**a**) Viability of 1.1B4 beta cells following their co-culture without pDCs (MC), with unstimulated pDCs, with DNA-LL37 complex stimulated pDCs [isolated from T1D (indicated as, ▲) and HC (indicated as, ●) subjects] and IFN-α. (**b**) Co-culture of 1.1B4 beta cells without monocytes (MC), with unstimulated monocytes, with DNA-LL37 complex stimulated monocytes [isolated from T1D (▲) and HC (●) subjects] and IFN-α. Beta cells were allowed to adhere overnight on the lower chamber of transwell plates and pDCs or monocytes stimulated with DNA-LL37 complexes (3 µg complex per 100 µL of media) were added to the upper chamber. IFN-α (2000 IU/mL) was used as positive control for contact independent apoptosis induction. Percent viability of beta cells was measured by Annexin V and PI assay. The data is presented as mean (±SEM) of six experiments. (**c**) Frequency of pDCs expressing IDO1 in T1D (n = 12) and HC (n = 10) subject groups both prior to and after stimulation with DNA-LL37 complexes. (**d**) MFI of IDO1 expressing pDCs in T1D and HC subject groups both prior to and after stimulation. FMO tube was used as negative control to gate positive cells. Relative gene expression of, (**e**) IDO1 in peripheral blood derived monocytes of T1D subjects (n = 15) compared to monocytes of healthy control subjects (n = 15). One-way ANOVA followed by Tukey’s multiple comparison test was used to compare the means and students’s T test was used for comparison in panel e. P  <  0.05 was considered statistically significant.

Stimulated effector cells (pDCs or monocytes) were separated from target cells (1.1B4 beta cells) by placing them in the upper chamber of a transwell insert for 24 hours, and the percent viability along with the proportion of apoptotic and necrotic beta cells were assessed by Annexin V and PI assay. We observed that the percent viability of beta cells co-cultured with stimulated pDCs or monocytes was significantly lower than with unstimulated pDCs (p = 0.03) or monocytes (p = 0.05) (Fig. 10a,b). Similarly, the stimulated pDCs or monocytes induced significantly higher apoptosis and necrosis of beta cells as compared with unstimulated pDCs (p = 0.03) or monocytes (p = 0.05) (Supplementary Figure S8). Upon comparing the T1D and HC groups, it was observed that the percent viability of 1.1B4 beta cell line was lower upon co-culture with stimulated pDCs or stimulated monocytes isolated from T1D subjects than those isolated from healthy subjects (p = 0.04) and (p = 0.05) respectively, whereas no difference was observed when unstimulated pDCs or monocytes were used. (Supplementary Figure S8).

### PDCs in T1D subjects express low levels of IDO1

Firstly, we stimulated monocytes and pDCs with DNA alone and DNA-LL37 complexes to compare the effects of their uptake on all subjects, the frequency of IDO1 + pDCs was found to be similar (p = 0.4) (Supplementary Figure S6).

Finally, to evaluate the tolerogenic capacity of pDCs, we assessed the expression of IDO1, the expression of which is used by pDCs to induce tolerance^48^, in the peripheral blood of T1D and HC subjects (Supplementary Figure S9). We observed decreased frequency of pDCs expressing IDO1 in the peripheral blood of the T1D subjects as compared to HC subjects (23.17 ± 3.04% vs 46.72 ± 5.86%) (p = 0.03). A similar trend was observed on assessing the MFI of IDO1 on pDCs as well (1704 ± 169 in T1D vs 3374 ± 370 in HC) (p = 0.002). Following the uptake of DNA-LL37 complexes, the frequency of IDO1 expressing pDCs was similar in T1D and HC subject groups (Fig. 10c,d).

### Monocytes in T1D subjects have reduced expression of IDO1

Monocytes can also acquire a tolerogenic phenotype by expressing IDO1 and gaining T cell suppressive activity^49^. Moreover, in comparison to mature monocytes or monocyte-derived DCs, monocytes by themselves produce a little amount of IDO1, hence qRT-PCR was used instead of flow cytometry for analysis due to its higher sensitivity in detecting low expression. The expression of IDO1 was also found to be reduced in monocytes of T1D subjects with a mean fold reduction of 0.20 ± 0.05 (mean ± SEM) (p = 0.0002; Fig. 10e) compared to HC subjects. These findings suggest that monocytes of T1D subjects have diminished tolerogenic capacity as compared to their normal healthy counterparts.

## Discussion

Initiation of autoimmune diabetes involves both inflammation and autoimmunity, the first being a result of the initial influx of immune cells such as macrophages, pDCs, conventional dendritic cells (cDCs) and neutrophils in the pancreatic islets. Evidence of the role of self-DNA can be further deduced from the fact that circulating DNA from beta cells has been detected in recent-onset T1D subjects^21^, along with increased neutrophilic activity, which may lead to the formation of DNA-immune complexes in the islets as also suggested by Diana *et al*. 2013^9^. In this work, we tried to get an imperative answer to the specific role that pDCs and monocytes could play during the initial stages of disease progression after their interaction with DNA-LL37 complexes. Our findings revealed a pathway by which self-DNA triggers IFN-α response upon binding with LL37 antimicrobial peptide indicating the possible role of DNA-LL37 complexes in creating beta cell-specific autoimmune responses via pDCs and monocytes.

The intracellular endosomal location of DNA receptors and the presence of DNase in serum and cytosol typically prevents the recognition of DNA-LL37 complexes by pDCs and monocytes^22^. Hence, we first demonstrated that LL37 was able to efficiently transport extracellular self-DNA into the cytosol of pDCs and monocytes by safeguarding DNA from lysis by nucleases. We were able to show that the transport of extracellular self-DNA into cytosolic compartments of monocytes and pDCs is enhanced by endogenous antimicrobial peptides like LL37, leading to induction of type I IFN. The role of LL37 becomes more important because DNA itself can also be internalized by pDCs and monocytes, but this does not happen naturally due to the presence of nucleases in intra- and extracellular environments including tissue fluids and plasma. This was also confirmed in our study where we observed significantly decreased uptake of DNA alone in the presence of human serum. The formation of DNA-LL37 complexes is driven by ionic interactions between the cationic residues of LL37 peptide and the anionic phosphate backbone of DNA^27^, and the uptake involves receptor-independent lipid raft-mediated endocytosis^43^. During T1D initiation, both pDCs and monocytes move towards pancreatic islets, but prior to exerting their proinflammatory phenotype, they must be activated. Our findings suggest DNA-LL37 complexes as activators of pDCs and monocytes, other than viral nucleic acids, which are accountable for IFN-α secretion during T1D pathogenesis in humans and NOD mice^50,51^. Direct evidence of interacting ligands of pDCs and monocytes infiltrating the islets are still lacking, hence we speculated that pDCs and monocytes might recognize self-DNA in complex with LL37 and are activated in the same manner even in the absence of viral infections.

To further support the claim that DNA-LL37 complexes and not DNA alone is required for the activation of pDCs and monocytes during T1D pathogenesis, we performed few experiments with DNA alone as a control along with DNA-LL37 complexes and observed the higher stimulatory potential of DNA-LL37 complexes in activating both pDCs and monocytes. This goes in conjunction with an important study by Lande *et al*. (2007) that LL37 converts DNA into a potent activator of pDCs and in the absence of LL37, the human DNA fails to induce IFN-α^27^. Additionally, there was no difference in the uptake of DNA-LL37 complexes by pDCs and monocytes of T1D and HC subjects in our study, which implies that the major difference lies not in the differential uptake but the way these cells of T1D and HC subjects sense DNA and get activated^52^.

Secondly, we observed that stimulation with DNA-LL37 complexes induces secretion of IFN-α by pDCs, the magnitude of which was found to be higher in subjects with T1D, indicative of the inflammatory nature of pDCs in these subjects. Several studies have focused on the role of IFN-α in the pathogenesis of T1D^8,10,53^. However, to our knowledge, it is the first study in humans investigating the pathological role of self-DNA in inducing increased expression of IFN-α in pDCs and monocytes in T1D subjects. Our assumption is based on other studies that have shown the effect of IFN-α *in situ* within the pancreatic islets^9^ since accessing islets during the development of disease in T1D subjects is not ethically and practically possible. Xia *et al*. (2014), found higher levels of serum IFN-α in subjects with T1D, source of which was suggested to be pDCs activated by persistent viral infection or self RNA/DNA released from damaged beta cells^54^. Our study suggests that in the absence of external factors like viral or bacterial nucleic acids, DNA-LL37 complexes might provide an initial stimulus leading to islet inflammation; however, more studies would be required to support its role. The process mediating the expression of IFN-α is already known, i.e. either through adaptor protein, stimulator of interferon genes (STING) and TANK binding kinase (TBK1) kinase signalling or through endosomal TLR9^55^. In monocytes as well, stimulation with DNA-LL37 complexes increased the expression of IFN-α in the majority of subjects. These results go in line with the previous report that monocytes secrete high levels of IFN-α (up to 600 pg/mL) in response to internalized genomic DNA and viral DNA^42^. The monocytes of the T1D group showed higher levels of IFN-α after stimulation, pointing towards the role monocytes could play in the initiation of inflammation during the initial stages of T1D. In support of these findings, a recent study by Zentsova *et al*. (2019), demonstrated that monocytes and pDCs participate in nucleic acid (microbial DNA and NETs containing self-DNA) recognition in T1D patients by involving TBK1 and STING intracellular DNA sensing receptors^52^. Contrary to our results, Merkle *et al*. (2015) have shown inhibition of pro-inflammatory responses after internalization of DNA by LL37 in non-immune cells like human microvascular endothelial cell^29^, which points towards the need of exploring the mechanisms behind differential behaviour of self-DNA in different cell types and conditions.

Once the role of DNA-LL37 complexes in the induction of IFN-α secretion by pDCs and monocytes was confirmed, we further interrogated whether this stimulation can induce maturation of these cells into active antigen presenters. Allen *et al*. (2009) have suggested that pDCs may stimulate islet-specific CD4 + T cells by capturing autoantibody antigen complexes and presenting peptides derived from these antigens through up-regulated MHC-II^56^. Similarly, we also observed an increase in HLA-DR expression along with co-stimulatory molecules CD80 and CD86 in stimulated pDCs, suggesting that pDCs can efficiently present antigens to CD4 + T cells during autoimmunity and may contribute to the pathogenesis of T1D. Conventionally, monocytes are not known for their antigen-presenting ability during T1D, but a study by Ren *et al*. (2017) has shown that intermediate monocytes in T1D patients have a better antigen presentation capability with higher levels of HLA-DR, CD86 and positive correlation with increased frequency of CD45RO + CD4 + memory T cells^14^. Therefore, our results become particularly interesting, as one of the major outcomes of DNA-LL37 complex stimulation on monocytes was an increase in the antigen presentation capacity, which can augment inflammatory responses in subjects with T1D by further activation of CD4 + T cells.

For a cell to become an efficient antigen presenter in addition to the required machinery, it must activate CD4 + T cells; classically, pDCs are not known for these skills. We have shown that following stimulation with DNA-LL37 complexes, pDCs can also act as autoantigen presenters. This is in line with earlier reports demonstrating that pDCs are able to present IA-2 peptides to CD4 + T  cells after short-term culture in the presence of IA-2 autoantibody+ serum during recent-onset T1D^56^. The antigen presentation by pDCs and monocytes may act as a stepping-stone in the generation of autoimmune responses since these cells are among the initial infiltrators of islets^9^. In T1D, the clearance of apoptotic cells is shown to be defective^57,58^, along with the flawed breakdown of extra-nuclear DNA in monocytes. pDCs can pick up the fragmented DNA bound to anti-nuclear antibodies during T1D pathogenesis in NOD mice^9,59^. The inflammatory and antigen-presenting phenotype is more consequential for beta cell destruction during T1D, probably due to the presence of beta cell-specific autoreactive T cells in circulation. The absence of autoreactive T cells could be a reason that renders any local inflammation developing into a full-blown autoimmune event during normal scavenging of self-DNA from all tissues including islets. In addition to the well-studied pro-inflammatory function, we also assessed the tolerance inducing capacity of pDCs and monocytes in terms of the expression of IDO1, which is known to induce Tregs and halt the proliferation of effector T cells in various autoimmune diseases^33,38,60^. Interestingly, in our study, we observed reduced expression of IDO1 in pDCs and monocytes of T1D subjects, which goes in conjunction with several reports that pDCs and monocytes in T1D are skewed towards pro-inflammatory phenotype^10,14^.

To further explore the effect of DNA-LL37 complex stimulation in inducing cytolytic phenotype in monocytes and pDCs, we cultured stimulated pDCs or monocytes with 1.1B4 beta cells in transwell plates and observed decreased viability of beta cells in the presence of stimulated pDCs or monocytes. These results go in conjunction with a previous report that activated macrophages cause *in vitro* apoptosis of beta cells^12^, although our study is the first to show that stimulation with DNA-LL37 complexes can enhance proinflammatory phenotype in the pDCs and monocytes. Similarly, we also demonstrated that pDCs stimulated with DNA-LL37 complexes could cause apoptosis of beta cells, via contact independent mechanisms. This also assigns an important role to the self-DNA leaking from apoptotic beta cells in the demise of healthy beta cells in the presence of pDCs and monocytes, which is likely to happen during the initial stages of T1D. Interestingly, we also observed that unstimulated pDCs and monocytes of T1D subjects also cause apoptosis of 1.1B4 beta cells, albeit lower than stimulated pDCs and monocytes, which could be attributed to the basal proinflammatory phenotype of these cells as observed in our study. Finally, we observed that the pDCs of T1D subjects have a higher capacity to induce apoptosis, suggestive of their possible role in inducing beta cell apoptosis during the early pathogenesis of T1D. Interestingly, we also observed some degree of apoptosis of 1.1B4 beta cells in response to IFN-α stimulation which is in contrast to previous reports that have shown the requirement of synergy between IFN-α with IL-1β to induce apoptosis in beta cell lines like EndoC-βH1^61^. Though we could not show any mechanistic details, our results do suggest further exploration of the role of IFN-α in the induction of apoptosis in beta cells from different sources.

To conclude, our results indicate that pDCs and monocytes can acquire an inflammatory phenotype upon stimulation with molecules like DNA-LL37 complexes. Therefore, pDCs and monocytes could play a role during the initiation of T1D, both by initiating inflammation through IFN-α and augmenting autoimmunity by activating CD4 + T cells. As the pDCs produce copious amounts of IFN-α and might provide the initial burst, further inflammation might then be sustained by the monocytes, as seen in other autoimmune diseases like SLE and psoriasis^42^. But unlike other disease targets, beta cells are known to be susceptible to IFN-α and as shown by other studies, there may be an induction of other irreversible inflammatory changes like long term MHC class I overexpression, ER stress and apoptosis^61,62^. The study thus warrants further research into the less explored area of genomic DNA complexes mediated innate cell responses, especially with CpG rich mitochondrial DNA and self-RNA during T1D initiation. The role of innate immune cells in T1D is undeniable^63^. Of late, the focus of immunotherapies is gradually encompassing targeting of innate immune cells, including monocytes^64^. Our study also asks for further research in applications of appropriate innate immune modulators like TLR antagonists to suppress activation of pDCs and monocytes or modulate them towards tolerogenic phenotype.

## Supplementary information

Supplementary information

## Data Availability

Data will be made available upon request.
