# Supplementary material for: Role of DNA-LL37 complexes in the activation of plasmacytoid dendritic cells and monocytes in subjects with type 1 diabetes
Source: Sci Rep. 2020 Jun 1;10:8896. doi: 10.1038/s41598-020-65851-y (PMC7264208; doi:10.1038/s41598-020-65851-y)
Supplement: Supplementary file 1 — Supplementary information [file 41598_2020_65851_MOESM1_ESM.pdf]

Role of DNA-LL37 complexes in the activation of plasmacytoid dendritic cells and monocytes in subjects with type 1 diabetes

Darshan Badal[1], Devi Dayal[1], Gunjan Singh[2], *Naresh Sachdeva[2], Departments of Pediatrics[1] and Endocrinology[2]. Post Graduate Institute of Medical Education and Research (PGIMER), Chandigarh, India.

***Corresponding Author:**

Naresh Sachdeva

Additional Professor

Department of Endocrinology,

Post Graduate Institute of Medical Education and Research (PGIMER),

Chandigarh - 160012, India.

Tel: +91-172-2755282, Fax: +91-172-2744401

*E-mail: naresh_pgi@hotmail.com

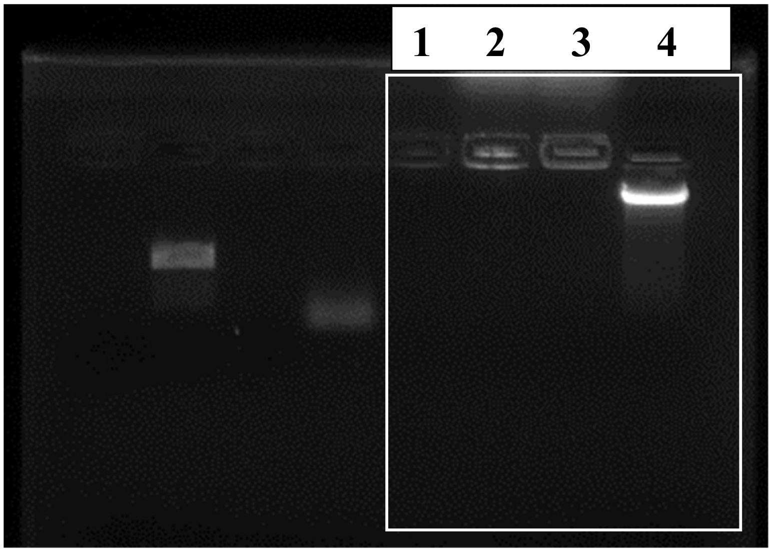

Figure S1: Complete image of the gel shown in figure 1a. Bands 1, 2, 3 and 4 (demarcated by the white box) were relevant for the experiment and hence were cropped to increase the clarity, conciseness and relevance in the manuscript.

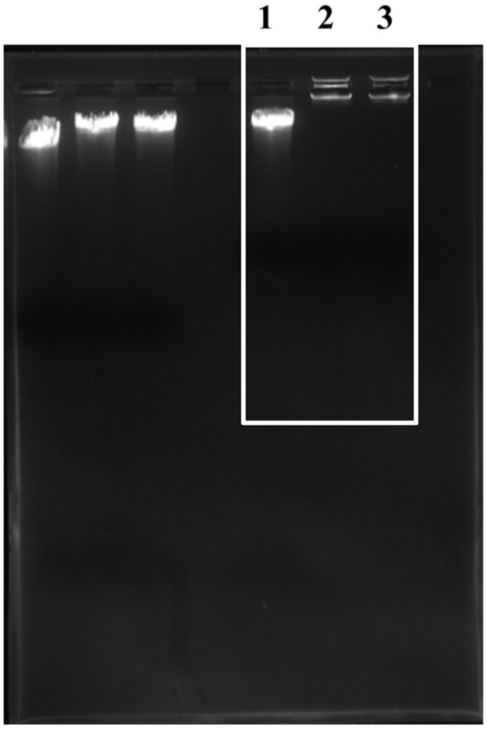

Figure S2: Complete image of the gel shown in figure 1b. Bands 1, 2 and 3 (demarcated by the white box) were relevant for the experiment and hence were cropped to increase the clarity, conciseness and relevance in the manuscript.

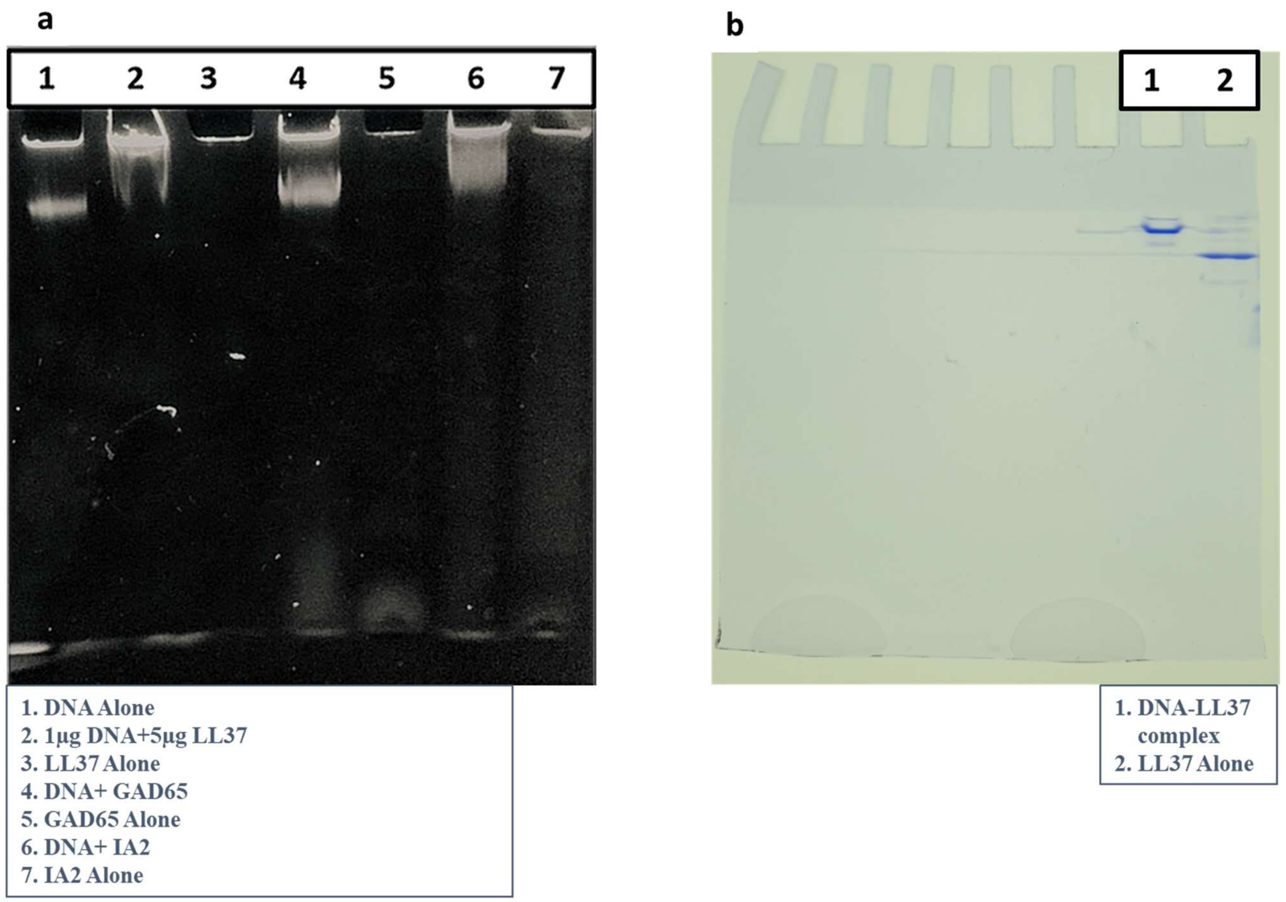

Figure S3: Complete image of the gel shown in Figures 1c and 1d. Fig a, Image of the gel was cropped horizontally to concise it. In figure b, bands 1 and 2 were relevant for the experiment and hence were cropped to increase the clarity, conciseness and relevance in the manuscript.

Supplementary figure: S4

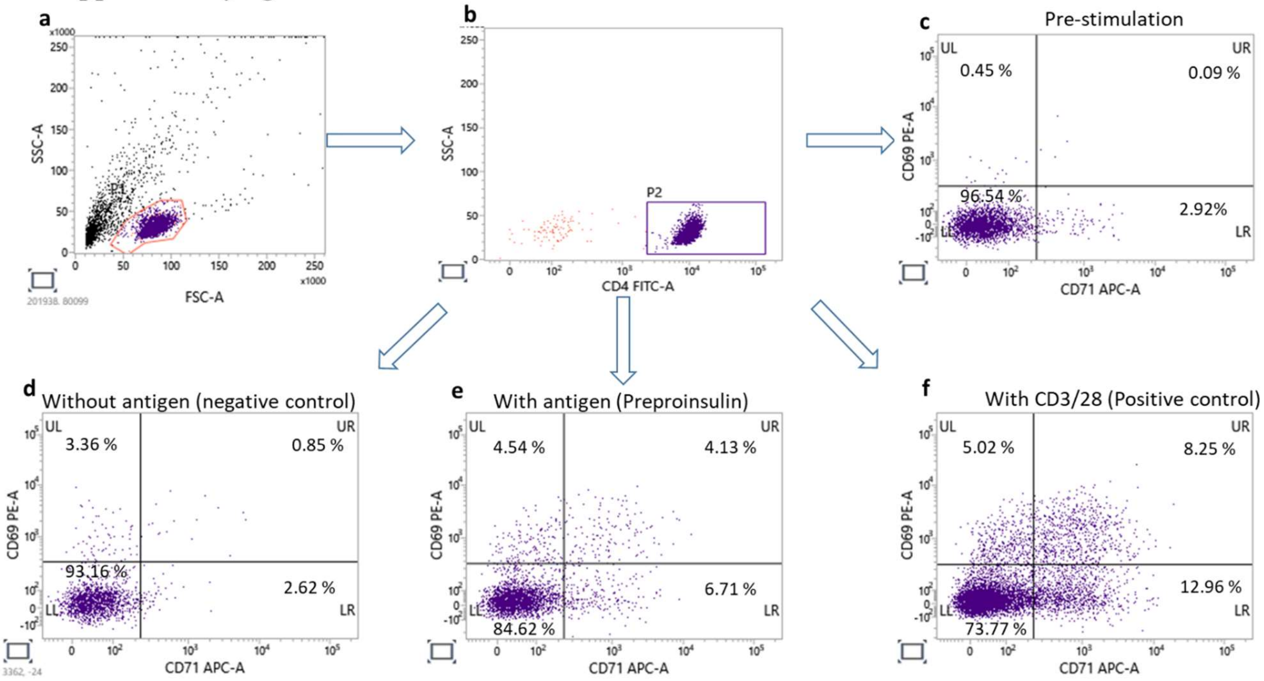

Figure S4: Representative flow cytograms showing CD4+ T cell stimulation using pDCs stimulated with DNA-LL37 complexes. a) Gating of PBMCs, b) Gating of CD4+ T cells, c) Pre-stimulation control, d) Negative control (without antigen). e) With antigen (preproinsulin), f) Positive control using both antigen and anti-CD3/CD28 antibodies.

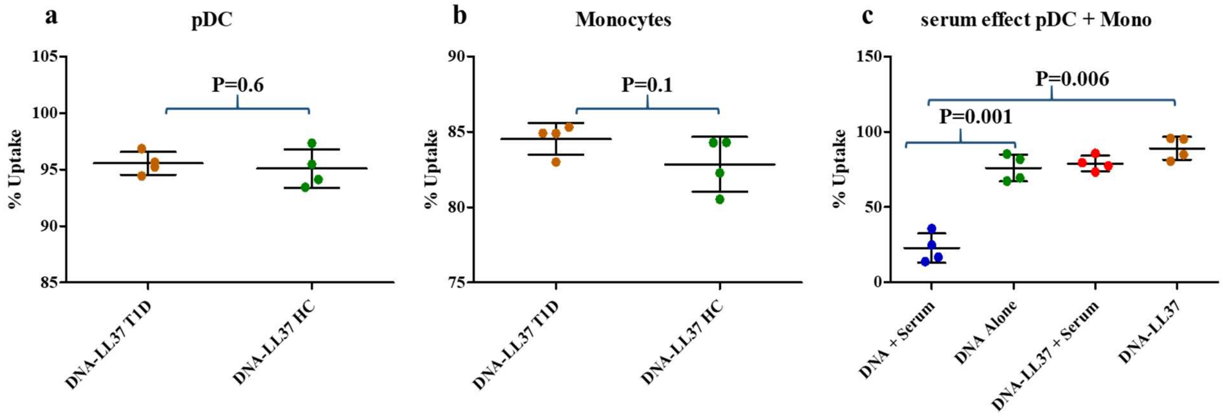

Figure S5: Uptake of DNA-LL37 complexes by, a) pDCs of T1D and HC subjects, b) monocytes of T1D and HC subjects. c) Uptake of DNA alone and DNA-LL37 complex by monocytes and pDCs in the presence and absence of autologous serum (combined data of pDCs and monocytes). The data is presented as mean (±SEM) of minimum four independent experiments. Students's T test was used for comparison in panel a and b, whereas one-way ANOVA followed by Tukey's multiple comparison test was used to compare the means in panel c. $P < 0.05$ was considered statistically significant.

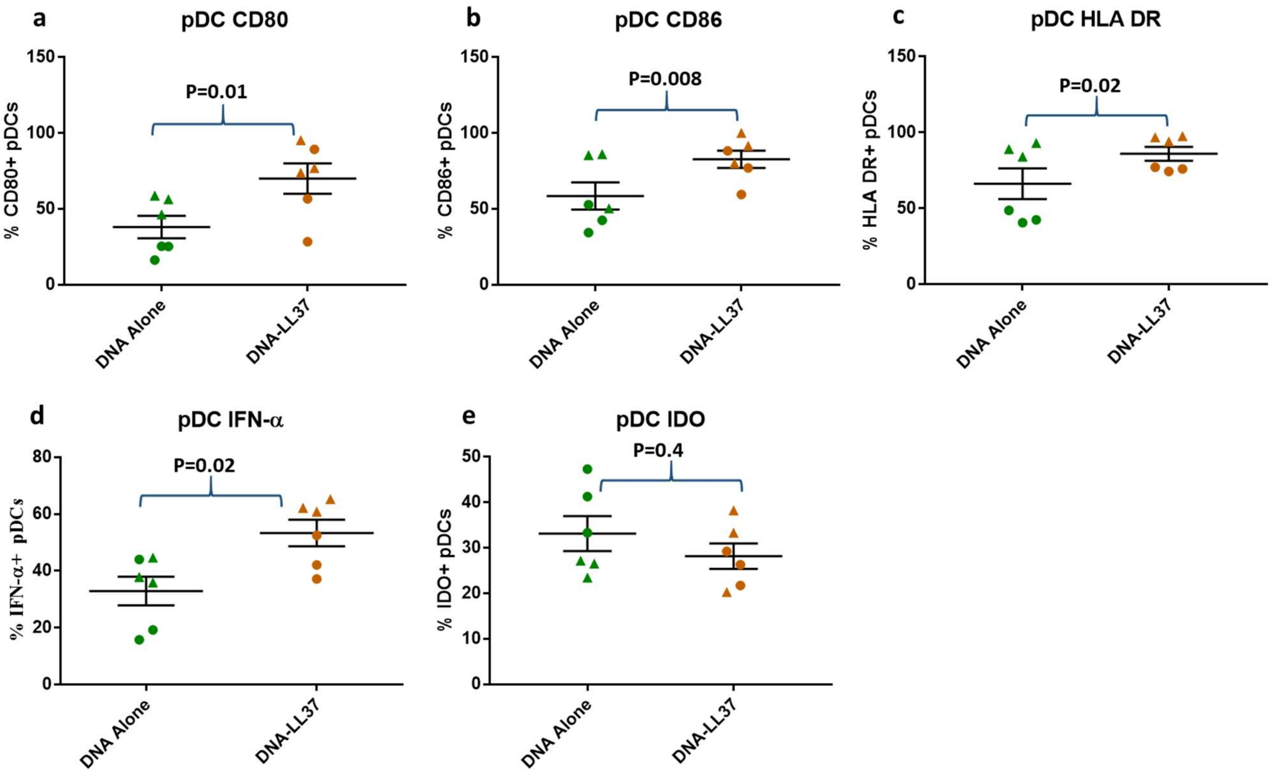

Figure S6: Comparison of DNA alone and DNA-LL37 complexes stimulation on the expression of activation markers of pDCs [isolated from T1D (indicated as, ▲) and HC (indicated as, ●) subjects, together].

Following stimulation with DNA alone (0.5 µg DNA per 100 µL of media) DNA-LL37 complex (3 µg complex per 100 µL of media), pDCs were compared for a) Frequency of CD80+ pDCs, b) Frequency of CD86+ pDCs, c) Frequency of HLA-DR expressing pDCs, d) Frequency IFN-α+ pDCs and e) Frequency IDO+ pDCs. Data is presented as mean (±SEM) frequency and students T-test was used to compare means. $P < 0.05$ was considered statistically significant. Relevant FMO tubes were used to set gates.

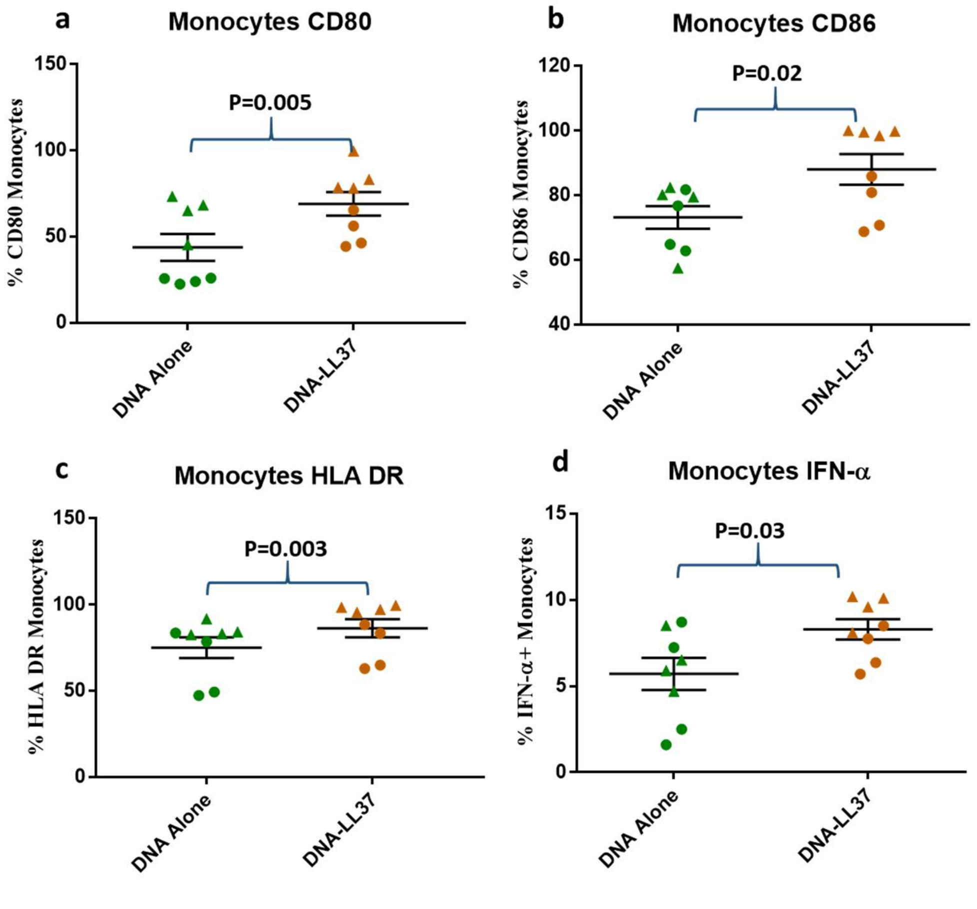

Figure S7: Expression of activation markers on the monocytes following uptake of DNA alone and DNA-LL37 complexes [isolated from T1D (indicated as, ▲) and HC (indicated as, ●) subjects, together].

Following stimulation with DNA alone (0.5 μg DNA per 100 μL of media) or DNA-LL37 complexes (3 μg complex per 100 μL of media), monocytes were compared for the expression of CD80, CD86, HLA DR and IFN-α. Frequencies of, a) CD80+ monocytes, b) Frequency of CD86+ monocytes, c) Frequency of HLA-DR+ monocytes and d) Frequency IFN-α+ monocytes. Data is presented as mean (±SEM). Students T-test was used to compare means. $P < 0.05$ was considered statistically significant. Relevant FMO tubes were used to set the gates.

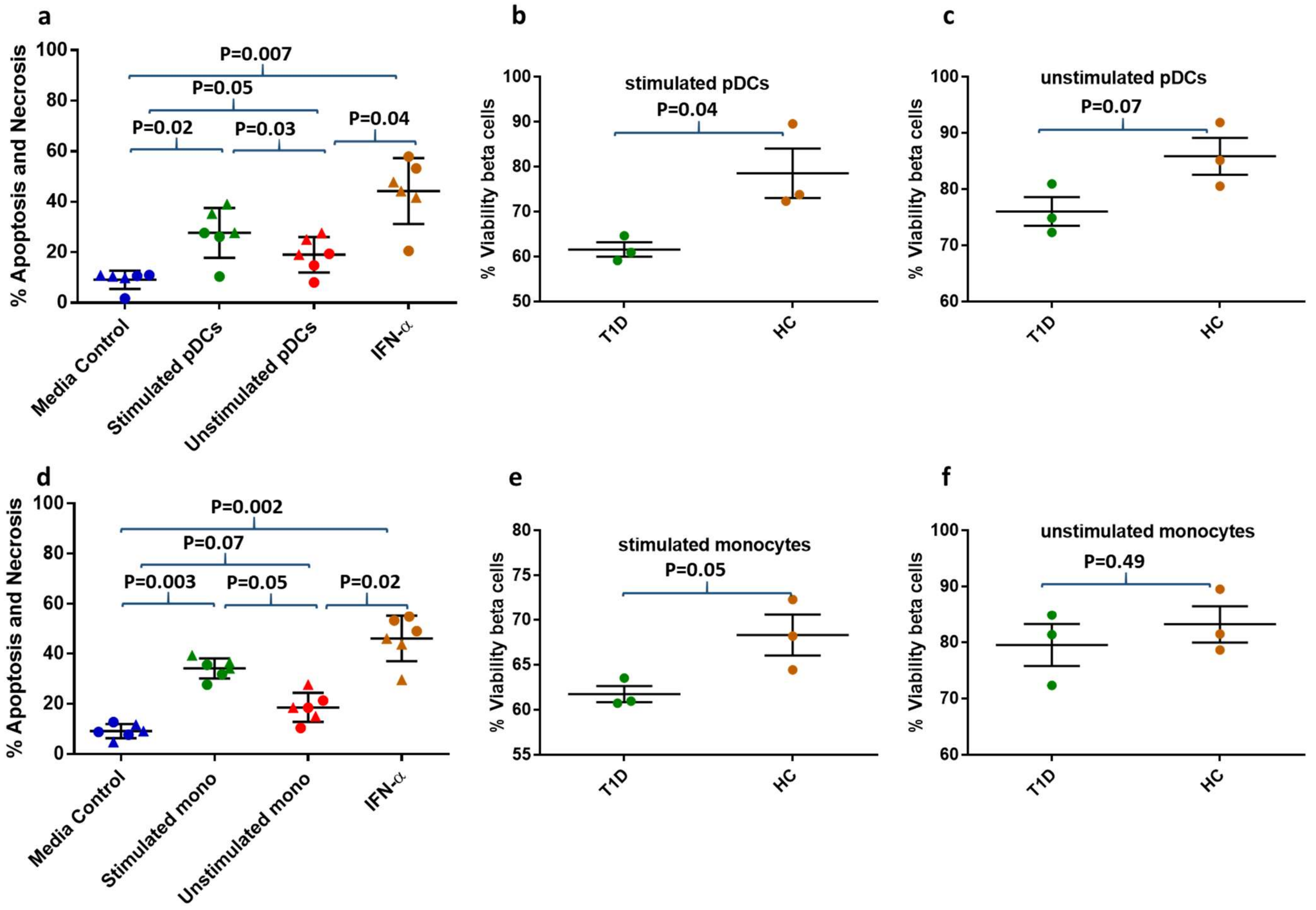

Figure S8: The percentage of apoptotic and necrotic beta cells and their percent viability after co-culture with pDCs and monocytes.

a) The percentage of apoptotic and necrotic 1.1B4 beta cells analyzed following their co-culture without pDCs (Media Control), with DNA-LL37 complex stimulated pDCs, with unstimulated pDCs, and IFN-α. b) Comparison of percent viability of 1.1B4 beta cells after co-culture with stimulated pDCs from T1D and HC subjects (n=3). c) Comparison of percent viability of 1.1B4 beta cells after co-culture with unstimulated pDCs from T1D and HC subjects (n=3). d) The percentage of apoptotic and necrotic 1.1B4 beta cells analyzed following their co-culture without monocytes (MC), with DNA-LL37 complex stimulated monocytes, with unstimulated monocytes and IFN-α. e) Comparison of percent viability of 1.1B4 beta cells after co-culture with stimulated monocytes from T1D and HC subjects (n=3). f) Comparison of percent viability of 1.1B4 beta cells after co-culture with unstimulated monocytes from T1D and HC subjects (n=3). Beta cells were allowed to adhere overnight on the lower chamber of transwell plates and pDCs unstimulated or stimulated with DNA-LL37 complexes (3 µg complex per 100 µL of media) were added to the upper chamber for 24 hours. IFN-α (2000 IU/mL) was used as positive control for contact independent apoptosis induction. Percent viability of beta cells was measured using Annexin V and PI staining by calculating the viable cells (Annexin V and PI negative by flow cytometry). Data points from T1D (indicated as, ▲) and HC (indicated as, ●) subjects are shown separately in panels a) and d). The data is presented as mean (±SEM) of six independent

experiments for panel a and d and of 3 independent experiments for panel b, c, e and f. One-way ANOVA followed by Tukey's multiple comparison test was used to compare the means for panels a and d, whereas students's T test was used for comparison in panel b, c, e and f. $P < 0.05$ was considered statistically significant.

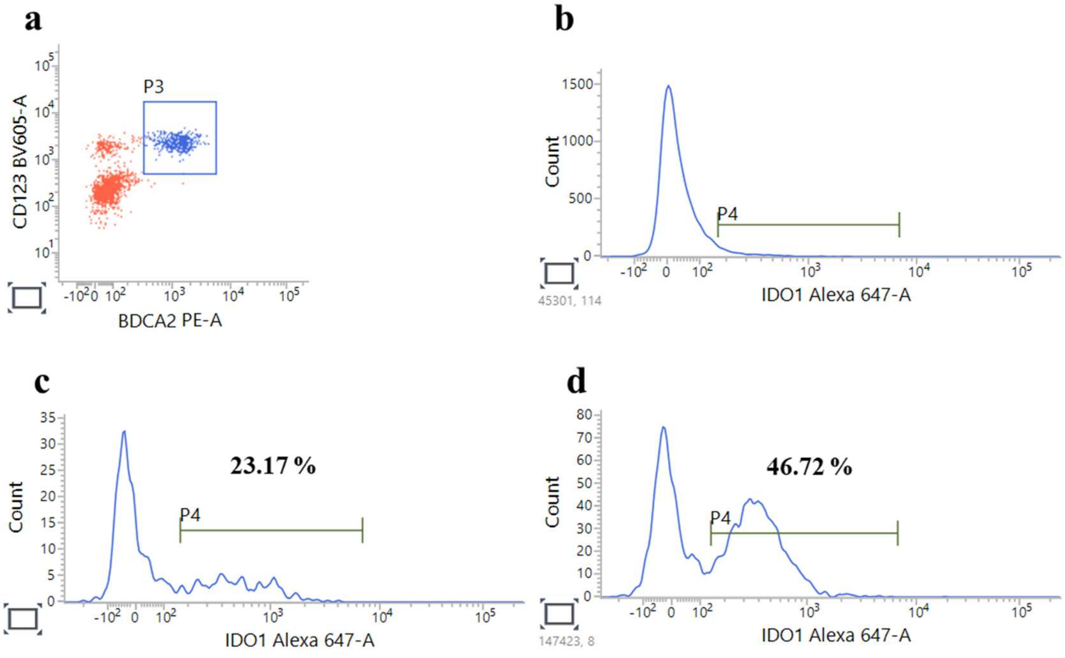

Figure S9: Representative flow cytograms showing the expression of intracellular IDO1 by pDCs prior to after stimulation with DNA-LL37 complexes. a) Gating of pDCs. b) Unstained control. Intracellular expression of IDO1 by pDCs, c) before and d) after stimulation with DNA-LL37 complexes.
